# Maturation of Heterogeneity in Afferent Synapse Ultrastructure in the Mouse Cochlea

**DOI:** 10.3389/fnsyn.2021.678575

**Published:** 2021-06-17

**Authors:** Shelby A. Payne, Matthew S. Joens, Heather Chung, Natalie Skigen, Adam Frank, Sonali Gattani, Kya Vaughn, Allison Schwed, Matt Nester, Atri Bhattacharyya, Guhan Iyer, Bethany Davis, Jason Carlquist, Honey Patel, James A. J. Fitzpatrick, Mark A. Rutherford

**Affiliations:** ^1^Department of Otolaryngology, Washington University School of Medicine, St. Louis, MO, United States; ^2^Center for Cellular Imaging, Washington University in St. Louis, St. Louis, MO, United States; ^3^TESCAN USA, Inc., Warrendale, PA, United States; ^4^Department of Biology, Washington University in St. Louis, St. Louis, MO, United States; ^5^Graduate Program in Audiology and Communications Sciences, Washington University School of Medicine, St. Louis, MO, United States; ^6^Department of Neuroscience, Washington University School of Medicine, St. Louis, MO, United States; ^7^Department of Cell Biology and Physiology, Washington University School of Medicine, St. Louis, MO, United States; ^8^Department of Biomedical Engineering, Washington University School of Medicine, St. Louis, MO, United States

**Keywords:** ribbon synapse ultrastructure, postsynaptic density, synaptic vesicles, modiolar, pillar, presynaptic density, FIB-SEM, developmental maturation

## Abstract

Auditory nerve fibers (ANFs) innervating the same inner hair cell (IHC) may have identical frequency tuning but different sound response properties. In cat and guinea pig, ANF response properties correlate with afferent synapse morphology and position on the IHC, suggesting a causal structure-function relationship. In mice, this relationship has not been fully characterized. Here we measured the emergence of synaptic morphological heterogeneities during maturation of the C57BL/6J mouse cochlea by comparing postnatal day 17 (p17, ∼3 days after hearing onset) with p34, when the mouse cochlea is mature. Using serial block face scanning electron microscopy and three-dimensional reconstruction we measured the size, shape, vesicle content, and position of 70 ribbon synapses from the mid-cochlea. Several features matured over late postnatal development. From p17 to p34, presynaptic densities (PDs) and post-synaptic densities (PSDs) became smaller on average (PDs: 0.75 to 0.33; PSDs: 0.58 to 0.31 μm^2^) and less round as their short axes shortened predominantly on the modiolar side, from 770 to 360 nm. Membrane-associated synaptic vesicles decreased in number from 53 to 30 per synapse from p17 to p34. Anatomical coupling, measured as PSD to ribbon distance, tightened predominantly on the pillar side. Ribbons became less spherical as long-axes lengthened only on the modiolar side of the IHC, from 372 to 541 nm. A decreasing gradient of synaptic ribbon size along the modiolar-pillar axis was detected only at p34 after aligning synapses of adjacent IHCs to a common reference frame (median volumes in nm^3^ × 10^6^: modiolar 4.87; pillar 2.38). The number of ribbon-associated synaptic vesicles scaled with ribbon size (range 67 to 346 per synapse at p34), thus acquiring a modiolar-pillar gradient at p34, but overall medians were similar at p17 (120) and p34 (127), like ribbon surface area (0.36 vs. 0.34 μm^2^). PD and PSD morphologies were tightly correlated to each other at individual synapses, more so at p34 than p17, but not to ribbon morphology. These observations suggest that PDs and PSDs mature according to different cues than ribbons, and that ribbon size may be more influenced by cues from the IHC than the surrounding tissue.

## Introduction

Auditory nerve fibers (ANFs) connect the ear to the brain. They are anatomically and functionally heterogeneous, but the relationships between anatomical and functional diversity are not completely understood. Each ANF is excited by a single ribbon synapse on an inner hair cell (IHC). Each IHC is frequency-tuned to a characteristic frequency (CF) and presynaptic to several ANFs (Liberman, 1980). Fibers with very similar or identical CF, likely excited by the same IHC or adjacent IHCs, can have very different spontaneous and evoked spike rates (Sachs and Abbas, 1974; Liberman, 1978; Jean et al., 2020). Each IHC is a sensory receptor driving synaptic release by a transmembrane potential that is graded over time and thought to be iso-potential due to the approximately cylindrical shape and relatively compact morphology of the IHC synaptic compartment (∼10 × 10 × 20 μm). Spontaneous (i.e., in silence) and sound-evoked spikes in ANFs rely on Ca^2+^-dependent exocytosis of glutamate from IHCs, evoking one spike per release event (Sewell, 1984; Robertson and Paki, 2002; Rutherford et al., 2012). Thus, IHC-afferent synaptic heterogeneities are posited to underlie the functional diversity of ANFs observed in one tonotopic location, at CF (Frank et al., 2009; Grant et al., 2010; Liberman et al., 2011; Wichmann and Moser, 2015; Rutherford and Moser, 2016). Synaptic sources of ANF diversity may arise from differences in the shapes and sizes of cytoplasmic ribbons, the presynaptic pool of vesicles they harbor, or the properties of the membrane densities containing presynaptic voltage gated Ca^2+^ channels and postsynaptic glutamate receptors.

Current knowledge about the relationships between IHC-afferent synaptic structure and ANF physiology come mainly from landmark studies in cat. There are interspecies similarities and differences in auditory nerve physiology which may or may not be related to synaptic anatomy. Some interspecies differences may be related to mechanisms of high-frequency vs. low-frequency hearing. In species with good low-frequency hearing like cat, guinea pig, chinchilla, and rabbit most ANFs have high spontaneous rate (SR) and low-threshold, while a smaller group of fibers has low-SR and hi-threshold (Liberman, 1978; Borg et al., 1988; Temchin et al., 2008; Furman et al., 2013). In contrast, animals like mice and rats that don’t hear well at low frequency (below 4 kHz, where phase-locking occurs and where response synchrony may encode stimulus strength) have compressed SR distributions that are not clearly bimodal and are shifted to lower SR (El Barbary, 1991; Taberner and Liberman, 2005). In gerbil and chinchilla, when SR for ANFs with CF less than or greater than 3 – 4 kHz are plotted, separately, the low-frequency SR distributions are clearly bimodal and the hi-frequency distributions are not (Ohlemiller and Echteler, 1990; Temchin et al., 2008). Differences between high- and low-frequency hearing may be correlated with structural differences between high- and low-frequency tonotopic regions, or between cochleae from animals that specialize in high- vs. low-frequency hearing.

Some observations of auditory nerve physiology and cochlear anatomy may transcend cochlear location or species, for example, the inverse relationship between SR and threshold (Liberman, 1978; Borg et al., 1988; Ohlemiller and Echteler, 1990; Taberner and Liberman, 2005; Temchin et al., 2008; Furman et al., 2013) and the modiolar-pillar gradient in synaptic ribbon size (Kantardzhieva et al., 2013; Michanski et al., 2019). Studies in cat and subsequently in many mammalian species established that ANFs fire action potentials at different SRs, and can be classified as low-SR (≤ 1 spike s^–1^) or hi-SR (> 1 spike s^–1^; summarized in Taberner and Liberman, 2005, their Figure 13). The SR, or rate of firing in the absence of an applied sound, is correlated with sound-response threshold: hi-SR fibers respond already to very soft sounds; low-SR fibers respond only to relatively loud sounds (Liberman, 1978). It was also established in cat that hi-SR fibers are thicker in caliber, have smaller presynaptic ribbons, and tend to be positioned on the pillar- or abneural-side of the IHC. Low-SR fibers are thinner, have larger presynaptic ribbons, and tend to be positioned on the modiolar- or neural-side of the IHC facing the spiral ganglion (Merchan-Perez and Liberman, 1996). The extent to which these structure-function relationships observed in cat generalize across mammalian species is unclear. To better understand how ANF diversity is achieved in a genetically tractable species widely used in auditory neuroscience, here we sought to better understand the ultrastructural maturation of cochlear ribbon synapses in C57BL/6J mice during late postnatal development with attention to position of innervation on the IHC.

## Methods

### Animals

Mice were studied in accordance with protocols approved by the Animal Studies Committee of Washington University in St. Louis. C57BL/6J background mice were obtained from The Jackson Laboratory. In this study we reconstructed three (IHCs) from one mouse aged p17, ∼3 days after the onset of hearing, and three IHCs from one mouse aged p34 when the cochlea is considered functionally mature.

### Tissue Processing and Embedding

Temporal bones were harvested, and cochleae were perfused via the round window with 0.15 M cacodylate buffer (pH 7.4) containing 2.5% glutaraldehyde, 2% paraformaldehyde, and 2 mM CaCl_2_. The cochleae were left in the cacodylate buffer fixative overnight at 4°C. The cochleae were then washed in cold cacodylate buffer containing 2 mM CaCl_2_. Without decalcification, the sample was then dissected to isolate pieces of the mid-cochlear organ of Corti from the surrounding bone using forceps and a ceramic blade. The staining procedure was modified from a previously described protocol (Deerinck et al., 2010). Briefly, the pieces were incubated in a solution containing 1.5% potassium ferrocyanide and 2% osmium tetroxide, 0.15 M cacodylate buffer with 2 mM CaCl_2_ on ice for 1 h. After this incubation, the sample was washed in double-distilled water (ddH_2_O), incubated in a filtered 0.1%thiocarbohydrazide (TCH) solution for 20 min at room temperature, washed in ddH_2_O again, and then placed in 2% osmium tetroxide for 30 min at room temperature. The samples were washed in ddH_2_O and incubated in 1% aqueous uranyl acetate at 4°C overnight, then incubated in 30 mM L-aspartic acid, 20 mM lead nitrate solution for 30 min in a 60°C oven, then washed again in ddH_2_O and dehydrated in dilutions (20, 50, 70, 90, 100, and 100%) of cold ethanol for 5 min each. After the dehydration step, the samples were left in ice-cold acetone for 10 min. For the embedding procedure, the samples were microwave irradiated (BioWave Pro, Ted Pella) at 300 W with 20 inches of Hg for 5 min prior to each incubation step. The samples were placed in a series of Durcapan ACM resin and acetone solution starting with 25% Durcapan:acetone for 2 h, 50% Durcapan:acetone for 2 h, and then 75% Durcapan:acetone for 2 h. Afterward, the cochleae were placed in 100% Durcapan for 2 h then embedded in fresh resin and baked at 60°C for 48 h.

### Focused Ion Beam Scanning Electron Microscopy

**FIGURE 1:**
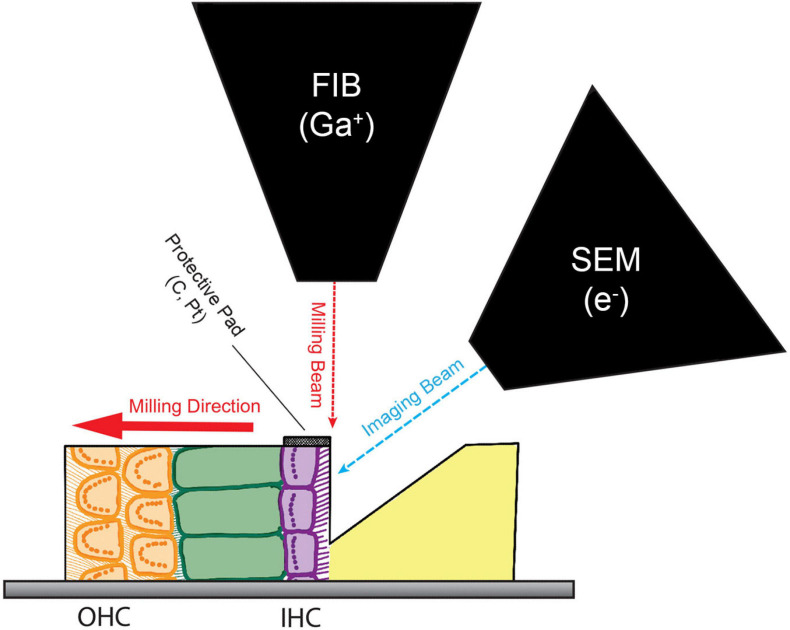
Block face imaging with Focused Ion Beam Scanning Electron Microscopy (FIB-SEM). Schematic of the dual beam method for serial block face imaging of inner hair cell (IHC) ribbon synapses. The milling beam and the imaging beam are offset by 52°. A protective pad made of carbon and platinum (C, Pt) was built over the IHCs by plasma-enhanced chemical vapor deposition using the gallium (Ga) FIB. The FIB was then used to bulk-trench the tissue in front of the IHCs, exposing these cells to the SEM. Images were acquired of this face at a resolution of 7 nm/pixel using backscatter detection. After collecting an image of the block face, the milling beam removed 7 nm of the sample from the surface before collecting the next image. This process was repeated until the sample was milled through the IHCs to the tunnel of Corti.

After embedding, we targeted approximately the 18 kHz region of the cochlea by facing the resin block with a diamond knife in a mid-modiolar radial orientation, verified by visualizing the tissue with a thick section containing organ of Corti and osseous spiral lamina. The sample was then loaded into a focused ion beam – scanning electron microscope (FIB-SEM; Crossbeam 540, Carl Zeiss; Figure 1) for 3D analysis as described previously (Narayan et al., 2014). In brief, the IHCs were located by imaging at 10 KeV with secondary electron detection. The region of interest was protected with a 30 μm × 20 μm platinum and carbon pad containing etched fiducial marks for focus and drift correction purposes. After capping, an approximately 50 μm deep trench was milled into the inner border cell region, along the tonotopic axis immediately to the modiolar side of the synaptic neuropil of the inner spiral plexus, using a 30 KeV gallium beam at 30 nA, followed by polishing at 7 nA at the same voltage. Images were then acquired of the newly formed block at 1.75 KeV and 1.2 nA using the Energy selective Backscattered (EsB) detector with a grid voltage of 1200 V, with 7 nm pixels, a dwell time of 3 μs and a line average of 3. Between each image acquisition, 7 nm of the block face was removed with the FIB using a 30 KeV, 1.2 nA beam.

### Image Processing and Segmentation

The collected images were then grayscale inverted, cropped, and aligned using the integrated Fibics software on the FIB-SEM. Images were collected with 7 nm isotropic voxels of the synaptic region from the bottom of the IHC nuclei to the habenula perforata, containing three IHCs from the modiolar-side facing the ganglion to the pillar-side facing the tunnel of Corti. The p17 raw data volume was cropped to a pixel format of 3,114 × 3,794 × 2,287 corresponding to approximately 22 × 27 × 16 μm in X, Y, and Z. The p34 raw data volume was cropped to 2,883 × 4,335 × 2,658 pixels corresponding to approximately 20 × 30 × 19 μm in X, Y, and Z. Before segmentation, the dataset was denoised using a non-local means filter with a sigma of 10 and a smoothing factor of 1 (Amira). Individuals familiar with ribbon synapse anatomy but blinded to mouse age and tissue orientation segmented all ribbons, presynaptic densities, postsynaptic densities, and the plasma membrane of the central IHC by hand using Amira software (Thermo Fisher). The IHC plasma membrane was segmented every 5–10 sections using the interpolation function. The first layer of synaptic vesicles surrounding the ribbon were marked manually in every 5th section (35 nm spacing) and counted using the Cell Counter Plugin in Image J. For movies of synaptic sub-volumes, image stacks were further aligned using the Linear Stack Alignment plugin (SIFT) in Image J. For display, fully segmented objects were smoothed for 20 iterations and a lamda (λ) = 0.6 to reduce the number of vertices, but maintain hard edges using the Surface Simplification Editor.

### Three-Dimensional Quantitative Analysis of Synaptic Structures

Segmented structures were analyzed in Amira using the Material Statistics module to obtain geometric measurements. We used Ferret Measures to determine the longest and shortest axes through the center of gravity of each synaptic ribbon. The long and short axes of the ribbons were not constrained to be orthogonal to each other. To measure the surface area of the ribbons, we used the Voxel Face Area module. For PDs and PSDs, we used Ferret Measures to determine the long and short axes through the center of gravity without constraining the measurements to be orthogonal. These measurements were then used to estimate surface area of the 3D projection on a 2D plane by modeling each synapse as an ellipse. To measure anatomical coupling between the PSD and the ribbons, we developed a workflow in Amira to measure the shortest distance to the ribbon surface from each vertex of each voxel comprising the ribbon-facing surface of the PSD, in order to construct histograms of proximity between the ribbon and the synaptic cleft. We used these ribbon-cleft proximity histograms as measurements of spatial coupling to compare sizes and shapes of synapses.

### Mapping Synapse Position

The XY imaging plane was similar to the orientation typically used when collecting optical sections in confocal microscopy of ribbon synapses in the organ of Corti whole-mount surface preparation (Khimich et al., 2005). The tonotopic dimension was oriented horizontally on the X axis. The habenular-cuticular (H-C) dimension of the IHC (from the basal synaptic pole to apical hair-bundle pole) was oriented vertically on the Y axis. The modiolar-pillar (M-P) dimension of the organ of Corti was traversed with sequential image sections along the Z axis. Synapse positions on the M-P and H-C axes were determined in two ways: (1) Native view - The Cartesian coordinates relative to the full range of positions in the tissue, with the most modiolar synapse and the most pillar synapse being equidistant from the origin at the center of the M-P axis, and the most habenular synapse defining the origin at the bottom of the H-C axis. In the native view, due to the relative positions of the cells within the tissue, synapses on the modiolar face of one IHC may reside in the same M-P position as synapses on the pillar face of an adjacent IHC. (2) Translated view – For each IHC, separately, the synapses were translated together along the H-C axis such that the most habenular synapse of each IHC was at the origin, and along the M-P axis such that the most modiolar synapse and the most pillar synapse of each IHC was equidistant from the origin at the center of the M-P axis. In the translated view, the synapses of each IHC were superimposed onto a common cell-centric reference frame, as illustrated in the text. In the native and the translated views, synapses were assigned as modiolar for coordinates < 0 or pillar for coordinates > 0 along the M-P (Z) axis.

### Statistical Analysis

Data sets were exported into Excel. All statistical values are presented as median ± standard deviation (SD) unless otherwise noted. All data sets were tested for normality using a Kolmogorov-Smirnov test with reference to a normal distribution. Wilcoxon rank-sum test (two-tailed) was used for pairwise comparisons between groups. Exact *p*-values are given. Effect size was calculated as Hedge’s *g* = difference between medians/pooled SD, where the pooled SD (*s*^∗^) is computed as

$$s^{*}=\sqrt{\frac{\left(n_{1}-1\right)\,s_{1}^{2}+\left(n_{2}-1\right)\,s_{2}^{2}}{n_{1}+n_{2}-2}},$$

where n_1_ is the number of units in group 1, n_2_ for group 2, s_1_ is the SD of group 1, and s_2_ for group 2. Spearman’s rho (ρ) was used to determine strength of association. Graphs labeled with rho has a significant *p*-value based on a two-tailed *t*-test. Graphs were plotted and statistics were performed in Igor Pro 7 (Wavemetrics).

## Results

Previous reports of cochlear synaptic morphology at the level of electron microscopy have focused mainly on embryonic and early postnatal development (Wong et al., 2014; Michanski et al., 2019), adult properties (Liberman, 1980; Merchan-Perez and Liberman, 1996; Slepecky et al., 2000; Kantardzhieva et al., 2013; Bullen et al., 2015), aging (Stamataki et al., 2006), or noise exposure (Bullen et al., 2019). Here, we focused on late postnatal development in unexposed wild-type C57BL/6J mice, from just after the onset of hearing function to maturation of cochlear function, which in mice happens from 2 – 4 weeks postnatal.

**FIGURE 2:**
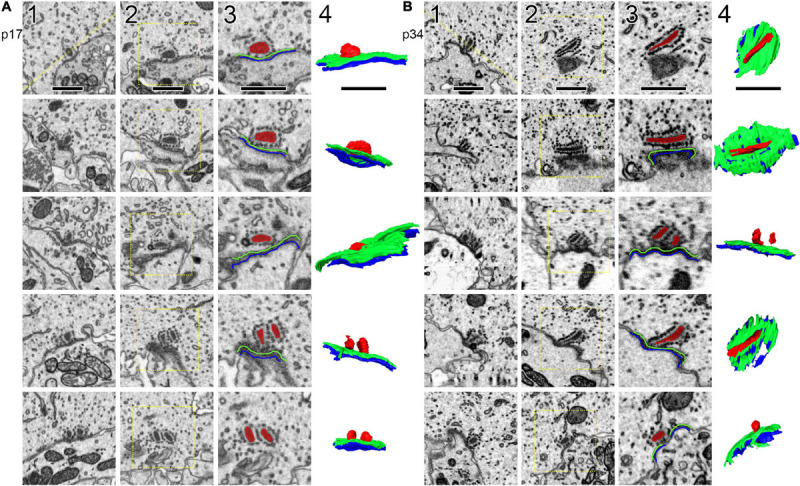
Segmentation of ribbons and membrane densities to generate 3-dimensional models of cochlear ribbon synapses. **(A)** Each of the 5 rows of images illustrates one ribbon synapse from the p17 sample. The volume of tissue surrounding each synapse was cropped in 3 dimensions. Column 1 shows images from the center of the stack, oriented with the presynaptic IHC on top, the postsynaptic afferent bouton on bottom, and the ribbon synapse in the center. The faint yellow dashed line in column 1 of row 1, passing through the center of the ribbon and parallel to the synaptic cleft, indicates the position of an orthogonal plane or section shown in column 2. The area inside the faint yellow dashed box in column 2 is enlarged in column 3, where the segmentation of synaptic structures is demonstrated. Column 4 contains a 2-dimensional representation of the 3-dimensional model generated from the series of segmented images. **(B)** Same as **(A)**, for the p34 sample. Red, presynaptic ribbon; green, pre-synaptic density; blue, post-synaptic density. Scale bars are 1 μm.

**FIGURE 3:**
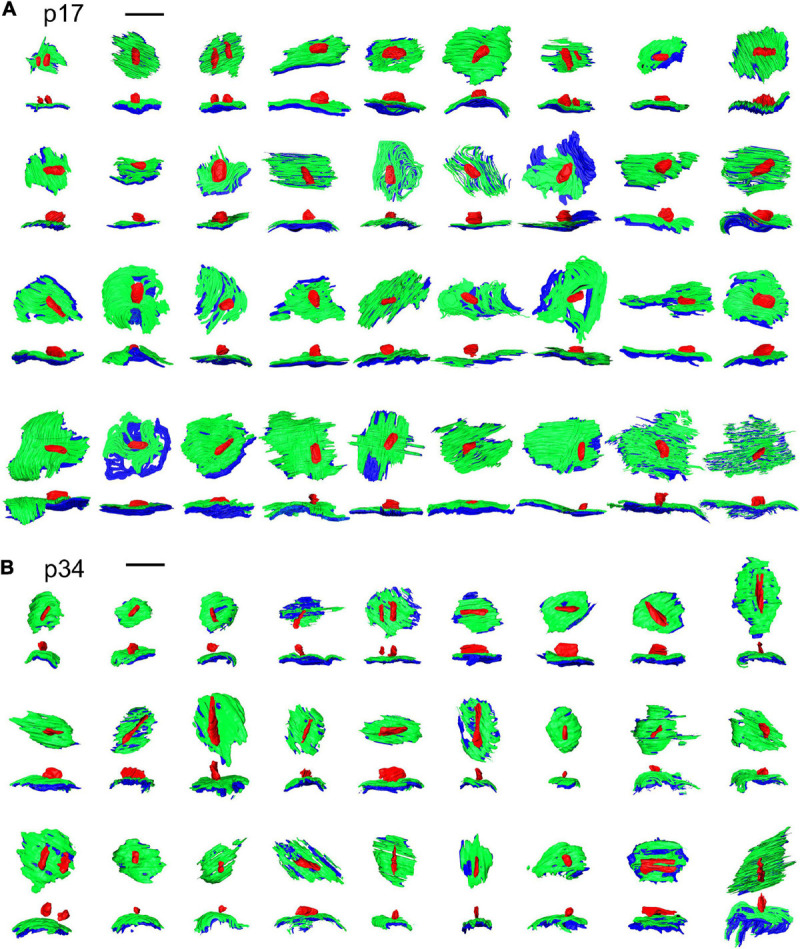
Ribbon synapse reconstructions from inner hair cells at p17 and p34. **(A)** 2-dimensional renderings of 3-dimensional reconstructions for p17 synapses. Each synapse is shown in, both, the en face or top-down view (upper) and a side view (lower). **(B)** Same as in **(A)**, for p34. Red, presynaptic ribbon; green, pre-synaptic density; blue, post-synaptic density. Scale bars are 1 μm.

Ribbons, presynaptic densities (PDs), and post-synaptic densities (PSDs) were segmented (Figure 2) along with the cytoplasm of the central IHC. PDs and PSDs were identified by a dark thickening of the membrane density on the surface of the IHC or the afferent bouton terminal, respectively (Supplementary Movie 1, 2). At p17 and p34, each bouton terminal contacting an IHC had a PSD juxtaposed to a PD. Each synaptic ribbon was anchored to a PD juxtaposed to a PSD. We did not observe any PD/PSDs without ribbons (i.e., no ribbonless synapses) nor any lone ribbons either anchored to the membrane or floating in the cytoplasm (i.e., no orphaned ribbons). The reconstructed volumes contained the basolateral compartment of one IHC in the center, plus parts of two adjacent IHCs. The p17 volume fully contained 38 synapses including 16 on the central IHC, plus 16 and 6 synapses on the two adjacent IHCs. Three of these synapses contained two ribbons each (i.e., double ribbons; Supplementary Movie 3). The p34 volume fully contained 32 synapses including 16 on the central IHC, plus 8 synapses on each of the two adjacent IHCs. Two of these synapses contained double ribbons. In our methodology the 7 nm pixel size in XY was matched by 7 nm resolution in the Z dimension, enabling visualization of the raw data in three orthogonal planes without anisotropic distortion (Supplementary Movies 4–6). In Figure 2, the raw data of several ribbon synapses is shown in both the original orientation, as the images were collected, as well as an orthogonal virtual section, demonstrating the benefit of isotropic voxels for 3-dimensional reconstructions. The reconstructed synapses from p17 and p34 are shown in the en face orientation and the side view in Figure 3.

### Trans-Synaptic Coordination of Morphological Maturation in the Membrane Densities

**FIGURE 4:**
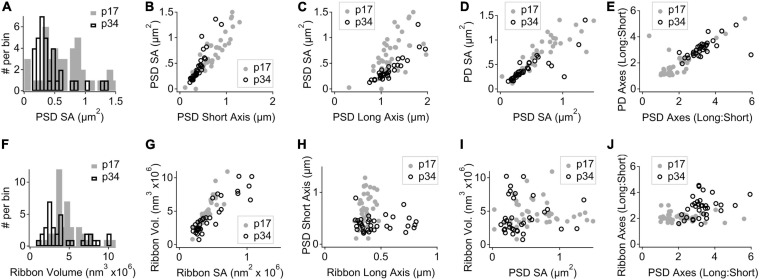
Morphological maturation of pre- and post-synaptic membrane densities and presynaptic ribbons. **(A)** Binned histogram of post-synaptic density surface areas (PSD SA) at p17 (filled gray bars) and p34 (hollow black bars). **(B)** Scatter plot of PSD SA vs. PSD short axis shows reduction in both metrics from p17 to p34 (SA, *p* = 6.5e^–4^; short axis, *p* = 7.9e^–6^; Wilcoxon). **(C)** PSD SA vs. PSD long axis shows that long axes are relatively unchanged between p17 and p34 (*p* = 0.97). **(D)** Pre-synaptic density (PD) SA vs. PSD SA for p17 and p34 (ρ = 0.90 and 0.87, respectively, Spearman’s Rho). **(E)** Long: short axis ratio of the PD vs. Long: Short axis ratio of the PSD shows positive correlation at both ages and an increase in both ratios from p17 to p34. **(F)** Binned histogram of synaptic ribbon volumes at p17 to p34, showing change in shape of distribution without significant difference of the mean (*p* = 0.07; Wilcoxon). **(G)** Scatter plot of ribbon volume vs. ribbon SA for p17 and p34. Neither changed significantly with maturation (*p* = 0.6). **(H)** PSD short axis vs. ribbon long axis. From p17 to p34, narrowing of the PSD short axis distribution and significant shortening (*p* = 7.9e^–6^) was accompanied by an increase in the upper range of ribbon long axes, although the increase in central tendency was not significant (*p* = 0.07). **(I)** Ribbon volume vs. PSD SA shows no clear relationship at p17 or p34. **(J)** Ribbon Long: Short axis ratio vs. PSD Long: Short axis ratio shows a developmental shift toward less spherical ribbons and less round PSDs from p17 to p34, but the degree of elongation on one side of the synapse is not well predicted by the degree of elongation on the other side.

For each PD and PSD we measured its longest axis, shortest axis, and surface area. From p17 to p34, the surface area (SA) of the median PSD decreased significantly by ∼ 50% (0.58 ± 0.36 vs. 0.31 ± 0.30 μm^2^), due to significant shortening of PSD short-axis by ∼ 50% (0.66 ± 0.29 vs. 0.36 ± 0.14 μm) while PSD long-axes were unchanged (1.12 ± 0.34 vs. 1.15 ± 0.42 μm^2^; Figures 4A–C). See Table 1 for group medians and ranges, as well as *p*-values and effect sizes of group comparisons. These differences in morphology of the PSD between p17 and p34 were accompanied by even larger developmental reduction in SA and short axis of the PD, on average (Table 1; SA effect sizes of 0.8 for PSD and 1.29 for PD). At p17, PDs were 29% larger than PSDs, while at p34 the SA of PDs and PSDs was nearly identical (0.33 ± 0.24 vs. 0.31 ± 0.29 μm^2^). At individual paired synapses, PD and PSD SA were positively correlated at both ages. The goodness of fit remained relatively stable from p17 to p34 with correlations of 0.90 and 0.87 respectively (Spearman’s rho), suggesting a mirroring of morphologies between paired pre- and post-synaptic membrane densities that was refined from p17 to p34 (Figure 4D). Synapses with greater long: short (L:S) axis ratios of the PD tended to have greater L:S axis ratios of the PSD, at both ages (Figure 4E). From p17 to p34, PDs and PSDs became less rounded. L:S axis ratios predominantly increased on both sides of the synaptic cleft, approximately doubling on average from p17 (median ratio: PD, 1.49; PSD, 1.69) to p34 (PD, 2.97; PSD, 3.19), again suggesting coordination of morphological maturation between pre- and post-synaptic membrane densities during late postnatal development.

**TABLE 1:** Median volume (nm^3^ × 10^6^), surface area (SA, μm^2^), axis length (nm), PSD to ribbon distance (nm), and number of vesicles per synapse at p17 and p34 by Modiolar-Pillar position in the translated view. For p17: *N* = 41 (23M/18P); p34: *N* = 34 (19M/15P).

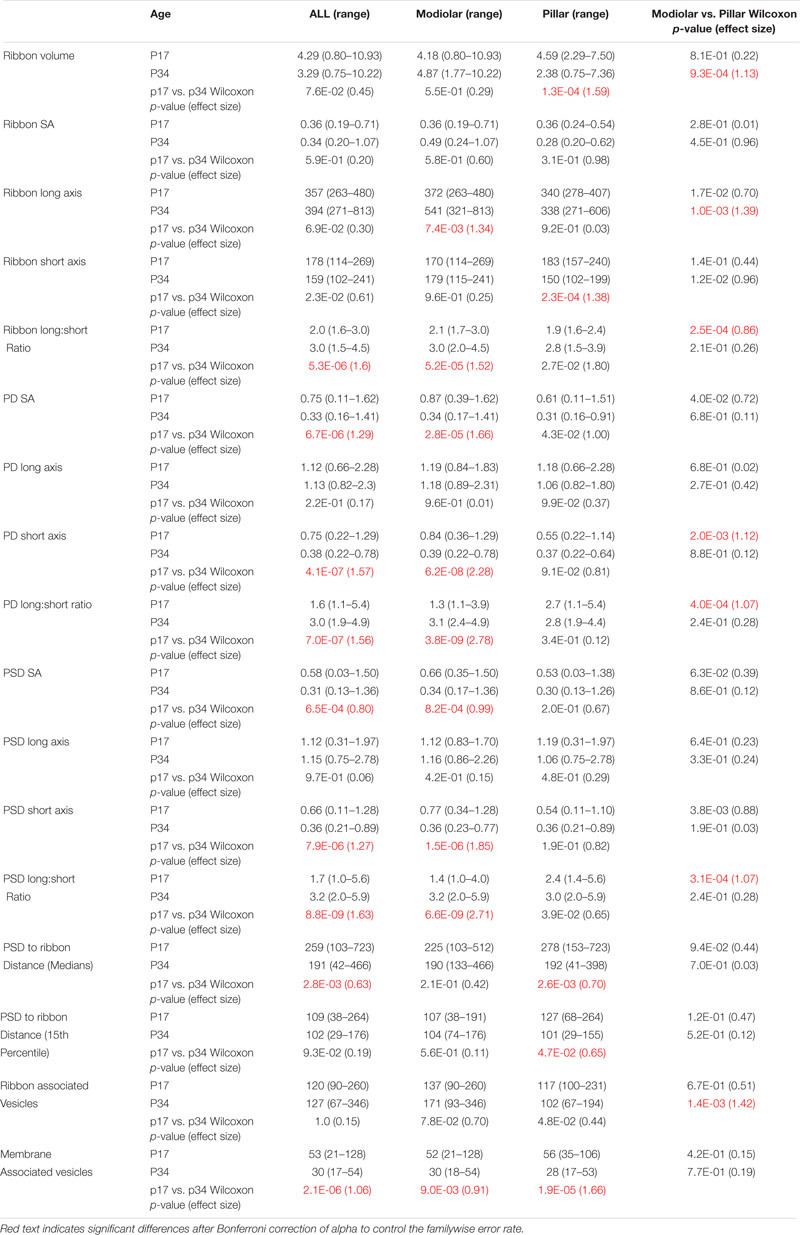

*Red text indicates significant differences after Bonferroni correction of alpha to control the familywise error rate.*

Presynaptic ribbons are amorphous ellipsoid-like cytoplasmic dense-bodies anchored to the active zone and decorated with glutamate-filled synaptic vesicles. For each ribbon we measured its volume, surface area, and the lengths of the longest and shortest dimensions through the center of mass (Table 1). On average, the p17 and p34 ribbon volumes were similar (4.3 ± 1.9 vs. 3.3 ± 2.6 e^6^ nm^3^), with an apparent shift to a bimodal distribution at p34 (Figure 4F). The plot of ribbon volume vs. SA deviated from the superlinear relationship defined by the geometry of a sphere, particularly at p34 when ribbons with the greatest SA had smaller volumes than expected from a linear trend (Figure 4G). This is consistent with a pattern of maturation in which some of the largest ribbons become less spherical as they elongate in one or two dimensions from p17 to p34. While the difference in ribbon SA between p17 and p34 was not significant, the median and the mean changed in opposite directions (median: 0.36 ± 0.11 vs. 0.32 ± 0.26 e^6^ nm^2^; mean: 0.39 vs. 0.46), consistent with a maturational process in which many ribbons become slightly smaller while a few ribbons become or remain larger. At p34, the ribbon long axes were broadly distributed relative to those at p17 (range: 263 – 480 at p17 vs. 271 – 813 nm at p34), again suggesting that some ribbons elongated with maturation, while PSD short axes became shorter on average and more narrowly distributed (range: 0.11 – 1.28 at p17 vs. 0.21 – 0.89 at p34; Table 1 and Figure 4H). Whereas the SA of the PD and PSD were clearly positively correlated (Figure 4D), there was no such correlation between ribbon volume and PSD SA (Figure 4I) or PD SA (not shown). Similarly, whereas the L:S axis ratios of the PD and PSD were clearly positively correlated (Figure 4E), there was no such correlation between the L:S axis ratios of the ribbon and the PSD (Figure 4J). Thus, in contrast to the apparent transsynaptic coordination between PDs and PSDs at p17 and p34, with maturation dominated by shortening of the short axes, ribbon morphology had no clear relationship to the PD or PSD at either age. In common, ribbons and membrane densities became less rounded with maturation from p17 to p34.

### Spatial Gradient of Ribbon Morphology Emerges With Cochlear Maturation

**FIGURE 5:**
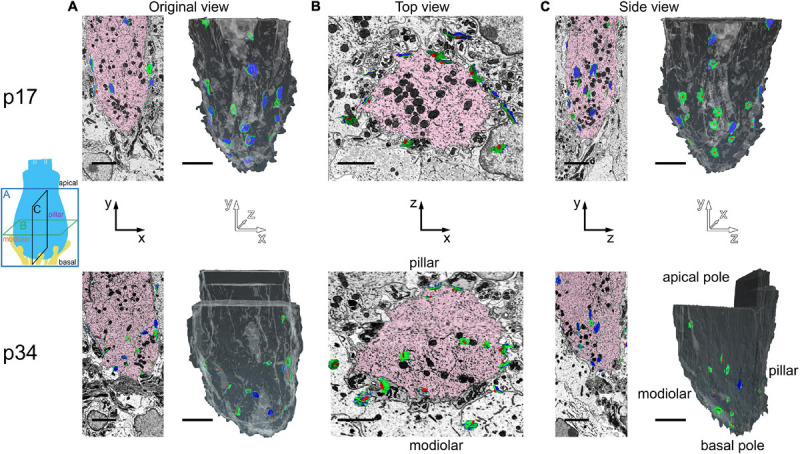
Inner hair cell segmentation and 3-dimensional reconstruction of ribbon synapse position in the organ of Corti. The diagram on the left indicates three orthogonal planes **(A, B,** and **C)** through the basolateral compartment of the IHC demonstrated in panels **A**, **B**, and **C**, respectively. The hair bundle is on the apical side (top) and the synapses are on the basal side (bottom). The cochlear spiral extends to the left and right. The modiolar-pillar axis is perpendicular to the page. **(A)** Original view–from the microscope perspective as the images were collected–for p17 (upper) and p34 (lower). On left, the central IHC (pink) is shown with segmented synapses overlaid on the raw data in the XY plane. On right, the IHC is colored in gray in a 2-dimensional rendering of the 3-dimensional reconstruction, oriented with the modiolar face in front. Red, presynaptic ribbon; green, pre-synaptic density (PD); blue, post-synaptic density (PSD). **(B)** The virtual XZ plane, showing the top-down view of the image in panel **(A)**. The modiolar side is on bottom and the pillar side is on top. **(C)** The virtual ZY plane, showing the side view of the image in panel **(A)**. Isotropic voxels enabled virtual sectioning in any orientation without distortion. Scale bars are approximately 3 μm.

**FIGURE 6:**
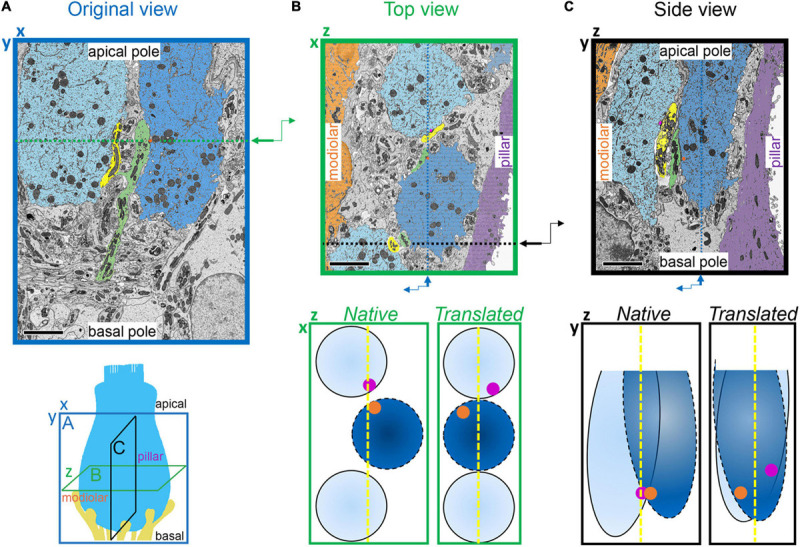
Translation of synapse positions to a common IHC reference frame. **(A)** Lower: Schematic indicating the three orthogonal planes demonstrated in panels **A**, **B**, and **C** (as in Figure 5). X-axis is the cochlear spiral; Y-axis is apical-basal; Z-axis is modiolar-pillar. Upper: Image acquired in the XY plane (original view) from the p17 sample. Two IHCs are colored light blue and blue; two afferent fibers are colored yellow and green; one synaptic ribbon is colored orange. Green dashed line indicates plane of virtual section displayed in panel **(B)**. **(B)** Upper: Virtual section indicated in panel A, showing top-down view in the ZX plane. Z-axis is the modiolar-pillar dimension. IHCs shifted to the modiolar side are light blue; IHCs shifted to the pillar side are darker blue; inner border cells are orange; inner pillar cells are violet. The terminal of the green fiber contacts the orange ribbon on the dark blue IHC. The terminal of the yellow fiber contacts a magenta ribbon on the light blue IHC. Black dashed line indicates plane of virtual section displayed in panel **(C)**. Lower, left: Schematic of IHCs in the native positions from the top view. Right: Translated positions, after aligning IHCs in the modiolar-pillar dimension. **(C)** Virtual section indicated in panel B, showing side view in the ZY plane. Lower, left: Schematic of IHCs in the native positions from the side view. Right: Translated view, after aligning IHCs in the modiolar-pillar and apical-basal dimensions. Scale bars are approximately 5 μm.

Next, we sought to measure spatial gradients in the sizes of ribbons, PDs, and PSDs, and to determine if the overall developmental changes tended to occur with a spatial preference. Each IHC resides in a position within the organ of Corti, and each synapse resides in a position on the IHC surface (Figures 5, 6, Supplementary Movies 7–10). A spatial gradient of decreasing ribbon size from the modiolar-side to the pillar-side of the IHC has been well-described with electron microscopy in the adult cat and developing mouse organ of Corti (Liberman, 1980; Merchan-Perez and Liberman, 1996; Kantardzhieva et al., 2013; Michanski et al., 2019). This modiolar-pillar (M-P) spatial gradient, also seen in confocal microscopy with antibodies to CtBP2/Ribeye, is typically quantified by splitting the synapses into two groups by position on either side of a plane dividing the modiolar-side from the pillar-side (Liberman and Liberman, 2016, 2015; Ohn et al., 2016). Although IHCs reside as a single row of cells located between the inner border cells (to the modiolar side) and the inner pillar cells (to the pillar side), sometimes the basolateral membranes of IHCs are not perfectly aligned along the M-P axis (Figure 6). Particularly in the mid-cochlea of the mouse, the basolateral membranes of neighboring IHCs tend to alternate relative positions along the M-P axis, which is apparent in top and side views of the dataset (Figures 6B,C). This IHC positioning in the tissue can cause some synapses on the modiolar face of one IHC to occupy the same M-P position in the tissue as synapses on the pillar face of a neighboring IHC. We therefore defined space in two ways (see section “METHODS”). In the “native” view, synapse position was calculated relative to the positions of all the other synapses in the region of imaging. In the “translated” view, synapse positions from each IHC were transformed onto a common, virtual central axis. In both cases, the middle point along the M-P axis (corresponding to the center of the tissue in the native view or the center of the IHC in the translated view) defined the position of the orthogonal plane dividing the modiolar (M) and pillar (P) groups.

**FIGURE 7:**
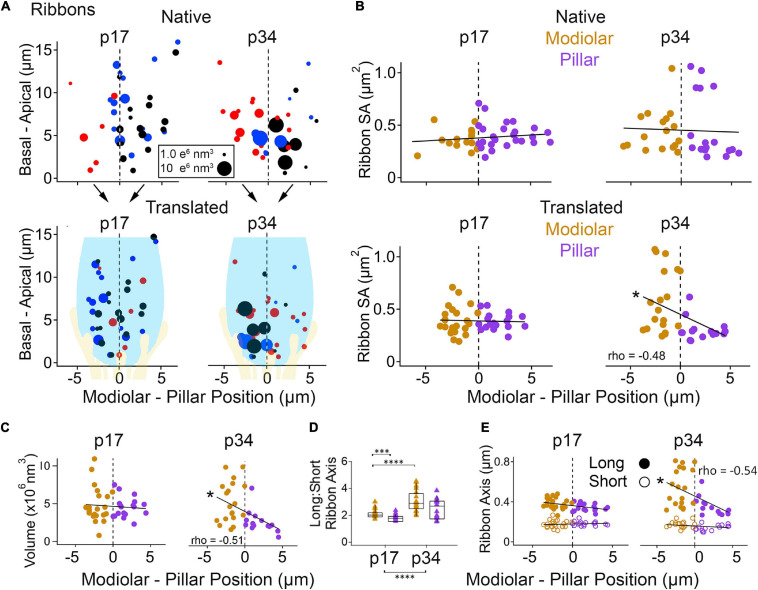
Ribbon morphology by modiolar-pillar position. **(A)** Synapse position along the basal – apical (or habenular – cuticular) axis of the IHC versus synapse position along the modiolar – pillar (M-P) axis of the organ of Corti (native view, upper) or of the superimposed IHCs (translated view, lower) for p17 (left) and p34 (right). Marker diameter is proportional to ribbon volume; marker color identifies the ribbon with one of 3 IHCs in the imaging region. In the translated view, a schematic of an IHC (light blue) and its afferents (light yellow) is overlaid to indicate the modiolar and pillar halves of the IHC. **(B)** Ribbon surface area versus M-P position in the native view (upper) or the translated view (lower) for p17 (left) and p34 (right). Gold and purple markers correspond to modiolar and pillar groups, respectively. Ribbon SA showed a significant M-P gradient in the translated view at p34 (*p* = 4.4E-03; rho = –0.48). **(C)** Ribbon volume versus M-P position in the translated view at p17 (left) and p34 (right). Ribbon volume showed a significant M-P gradient at p34 (p34: *p* = 6.4E-11; rho = –0.51). **(D)** Ribbon Long axis: Short axis ratios for modiolar (gold) or pillar (purple) ribbons in the translated view for p17 and p34. Within each box, horizontal line denotes the median; box extends the interquartile range; vertical line denotes the 10-90 percentile range. Significant difference in ratios over development (*p* = 5.3E-06) came mainly from significant changes on the modiolar side (*p* = 5.2E-05). **(E)** Ribbon long axis (filled circles) and short axis (open circles) versus M-P position in the translated view for p17 (left) and p34 (right). The ribbon long axis showed a significant M-P gradient at p34 (*p* = 1.1E-03; rho = –0.54). *p < 0.05, ***p < 0.001, ****p < 0.0001.

In the native view, the M-P axis ranged over approximately 14 μm (Figures 7A,B, upper subpanels). In the translated view, the M-P axis ranged over the width of one IHC, approximately 8 μm (Figures 7A,B, lower subpanels). At p17 there was no apparent gradient of ribbon SA along the M-P axis in either the native or translated views (Figure 7B, left subpanels), and there was no significant difference in SA between modiolar and pillar ribbons (Table 1, translated view; Supplementary Table 1, native view). At p34, a M-P gradient of ribbon SA was apparent in the translated view but not in the native view (Figure 7B, right subpanels; significant linear trend, Spearman’s rho = -0.48). Comparisons of SA between modiolar and pillar ribbons did not reach statistical significance even at p34 in the translated view, in which the median modiolar ribbon had nearly twice the SA of the median pillar ribbon (Table 1; 0.49 ± 0.29 vs. 0.28 ± 0.1 μm^2^). Modiolar ribbon volume was significantly larger than pillar ribbon volume only at p34 in the translated view (4.9 ± 2.6 vs. 2.4 ± 1.5 μm^3^), as pillar-side ribbons became significantly smaller from p17 to p34 (*p* = 1.3e^–4^) while modiolar-side ribbon volumes were unchanged (*p* = 0.55; Table 1 and Figure 7C).

The ribbon long-axis was significantly longer in the modiolar group than the pillar group at p17 (*p* = 1.7e^–2^, effect size = 0.7), and more so at p34 with larger effect size (*p* = 1.0e^–3^, effect size = 1.39) due to lengthening of the long-axis specifically on the modiolar side from p17 to p34 (372 ± 52 vs. 541 ± 179 nm; Table 1, Figures 7D,E), with synapses in the translated view. In the native view, this comparison was significant only at p34 (Supplementary Table 1 and Supplementary Figures 1A,B). The range of long axes increased 3-fold as the upper end was extended from p17 (263 – 480 nm) to p34 (271 – 813 nm). Ribbon short-axis was significantly longer in the modiolar group than the pillar group only at p34 in the translated view (*p* = 1.2e^–2^, effect size = 0.96) after significant shortening of the short-axis on the pillar side from p17 to p34 (183 ± 20 vs. 150 ± 28 nm, *p* = 2.3e^–4^, effect size = 1.38).

To determine if there were gradients in synapse size along the dimension from the basal pole of the IHC (near the habenula perforata) to the apical pole of the IHC (near the cuticular surface of the organ of Corti), we plotted the data as a function of habenular-cuticular position. There was no apparent spatial gradient of ribbon axes at p17 in either the native or translated view (Supplementary Figures 2A,C). However, an increasing habenular-cuticular gradient did emerge at p34 in the translated view for ribbon long axes (Supplementary Figure 2C), although it was not a significant correlation nor was there a significant difference between habenular and cuticular groups (*p* = 0.65). In conclusion, ribbon long axes increase over development, predominantly on the modiolar side, while ribbon short axes became shorter on the pillar side. Orthogonal to the M-P axis, the ribbons with the longest long-axes were located in the cuticular half of the distribution of synapses, toward the apical part of the IHC, placing the longest ribbons in the modiolar/cuticular quadrant.

### Spatial Gradient of PSD Morphology Diminishes With Cochlear Maturation

**FIGURE 8:**
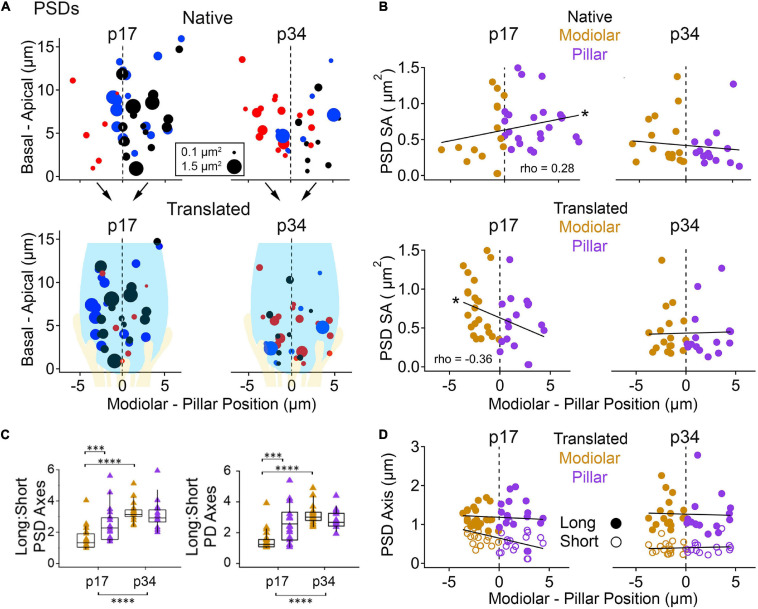
Post-synaptic density (PSD) morphology by modiolar-pillar position. **(A)** Synapse position along the basal – apical (or habenular – cuticular) axis of the IHC versus synapse position along the modiolar – pillar (M-P) axis of the organ of Corti (native view, upper) or of the superimposed IHCs (translated view, lower) for p17 (upper) and p34 (lower). Marker diameter is proportional to PSD surface area; marker color identifies the PSD with one of 3 IHCs in the imaging region. In the translated view, a schematic of an IHC (light blue) and its afferents (light yellow) is overlaid. **(B)** PSD surface area versus M-P position in the native view (upper) or the translated view (lower) for p17 (left) and p34 (right). Gold and purple markers correspond to modiolar and pillar groups, respectively. Significant gradients were observed at p17 in native (p = 7.6E-02; rho = 0.28) and translated views (*p* = 1.9E-02; rho = –0.36). **(C)** PSD (left) and PD (right) Long axis: Short axis ratios for modiolar (gold) or pillar (purple) ribbons in the translated view for p17 and p34. Significant difference in the PSD Long: Short axis ratio over development (*p* = 8.8E-09) came from changes on the modiolar side (*p* = 6.6E-09). Significant difference in the PD Long: Short axis ratio over development (*p* = 7.0E-07) came from changes on the modiolar side (*p* = 3.8E-09). **(D)** PSD long axis (filled circles) and short axis (open circles) versus M-P position in the translated view for p17 (left) and p34 (right). *p < 0.05, ***p < 0.001, ****p < 0.0001.

In Figure 8, we treated PSDs the way we treated ribbons in Figure 7. As seen in Figure 4 and Table 1, the SA of the PSD and the PD are significantly reduced from p17 to p34. In the native view, this reduction was significant on the pillar side only (Supplementary Table 1). In the translated view, this developmental reduction in SA was predominantly on the modiolar side (Table 1) for the PD (0.87 ± 0.36 vs. 0.34 ± 0.28 μm^2^) and the PSD (0.66 ± 0.35 vs. 0.34 ± 0.29 μm^2^). At p17, a significant spatial gradient in PSD SA in the native view (increasing modiolar to pillar, Spearman’s rho = 0.28) was reversed in the translated view (decreasing modiolar to pillar, Spearman’s rho = −0.36, Figure 8B, left subpanels), but modiolar-side PSDs were not significantly different from pillar-side PSDs in either view. Differences were slightly larger for PDs than PSDs, reaching marginal significance only at p17, and in opposite directions in the native and translated views like the PSDs (Table 1 and Supplementary Table 1; *p* = 0.045 and 0.04, respectively).

At p34, modiolar side PDs and PSDs had very similar SA, on average, as pillar side PDs and PSDs, in either view (Figure 8B, right subpanels; overall range 0.16 – 1.4 μm^2^). For the 8 groupings (PD or PSD, modiolar or pillar, native or translated) the medians ranged 0.30 – 0.34 μm^2^, *p* = 0.53 – 0.86. The overall reduction in SA of PDs and PSDs from p17 to p34 was associated with a significant shortening of the short-axes of the membrane densities, not the long axes (Figures 4B,C; Table 1). This reduction in PD and PSD short axes occurred on the pillar side in the native view (Supplementary Figure 1C) and on the modiolar side in the translated view (Figures 8C,D). However, the modiolar vs. pillar difference in short axes at p17 was significant only in the translated view for the PD (0.84 ± 0.24 vs. 0.55 ± 0.28 μm, *p* = 2e^–3^) and the PSD (0.77 ± 0.26 vs. 0.54 ± 0.26 μm, 3.8e^–3^). Unlike presynaptic ribbons in which the variance of long: short axis ratios increased with lengthening of the long axis on the modiolar side from p17 to p34 (Figures 7D,E), the variance of long: short axis ratios of PDs and PSDs decreased from p17 to p34 with shortening of the short axis on the modiolar side (Figures 8C,D). Along the habenular-cuticular dimension, there was no apparent spatial gradient of axis length at p17 or p34 in either the native or translated view (Supplementary Figure 2B,D). In conclusion, pre- and post-synaptic membrane densities grow narrower over the course of development, predominantly on the modiolar side of the IHC as seen in the translated view (Figure 8D and Table 1).

### Spatial Dependence of Pre- and Post-Synaptic Relationship

**FIGURE 9:**
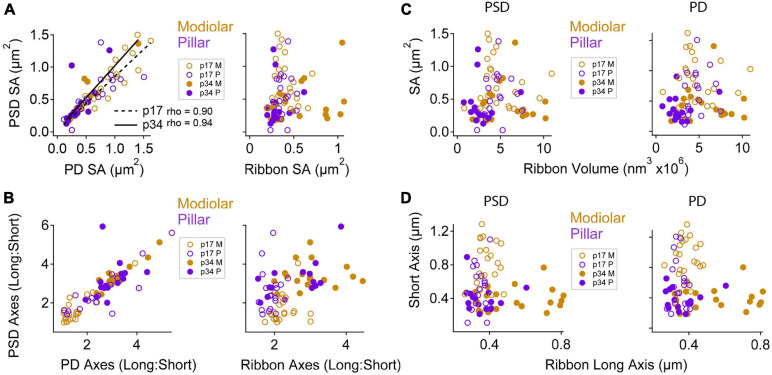
Maturation of pre- and post-synaptic morphological relationships by modiolar-pillar position. **(A)** PSD SA vs. PD SA (left) or Ribbon SA (right). The correlations between PSD SA and PD SA were significant at p17 (*p* = 3.7E-03; rho = 0.9) and p34 (*p* = 3.3E-03; rho = 0.94). In all panels: open circles are p17, filled circles are p34; gold circles are modiolar, purple circles are pillar. **(B)** PSD L:S axis ratio vs. PD L:S axis ratio (left) or Ribbon L:S axis ratio (right). **(C)** PSD SA (left) or PD SA (right) vs. Ribbon volume. **(D)** PSD short axis (left) or PD short axis (right) vs. Ribbon long axis.

Next, we examined the relationships between ribbons, PDs, and PSDs at paired synapses with respect to modiolar and pillar groups. From herein, we refer to the translated view only, unless otherwise noted. PD and PSD surface areas tended to be very similar at individual synapses (Figure 9A, left; Spearman’s rho = 0.90 at p17, and 0.94 at p34 with the removal of one outlier). The slopes of the linear fits were: p17 modiolar, 0.89; p17 pillar, 0.69; p34 modiolar, 0.97; and p34 pillar, 1.1. As the SA of PDs and PSDs became smaller with maturation, the long: short axis ratios became larger. These changes were largest for the modiolar synapses between p17 and p34 because the modiolar group at p17 had smaller long: short ratios than the pillar group (Figure 9B), suggesting the pillar-side synapses had a more mature morphology at p17 than the modiolar-side synapses. Although the relationships between SA of PDs and PSDs were well-described by linear trends, the SA of the ribbon was not a good predictor of PSD SA (Figure 9A, right).

We plotted the long: short PSD axis ratios vs. the long: short ribbon axis ratios and did not see any clear relationship between ribbon shape and PSD shape for modiolar or pillar synapses at p17 (Figure 9B, right). For example, while the pillar-side PSDs had more mature-like morphologies than the modiolar-side PSDs at p17 (i.e., larger L:S ratios), the pillar-side ribbons did not (Figure 9B, right), suggesting that PDs may take maturational cues from the PSD (or vice versa) rather than the ribbon. Similarly, there was no clear systematic variation in morphology of the PSD or the PD with ribbon volume (Figure 9C). The variation within or across the four groups of PDs or PSDs was not well described by linear functions of ribbon size. To ask if there was coordination between ribbon elongation and narrowing of the membrane densities over postnatal maturation, seen particularly on the modiolar side, we explored the relationship between PSD short axis and ribbon long axis (Figure 9D). Although the largest ribbons tended to be on the modiolar side of p34 IHCs, those ribbons were not paired with particularly large PDs or PSDs (Figure 9D). In conclusion, the apparent coordination of morphology between pre- and post-synaptic membrane densities did not appear to extend to the ribbon, suggesting separate IHC and neuronal cues for maturation of synaptic heterogeneity over late postnatal development.

### Pillar-Side Maturation in Spatial Coupling Between the Ribbon and PSD

**FIGURE 10:**
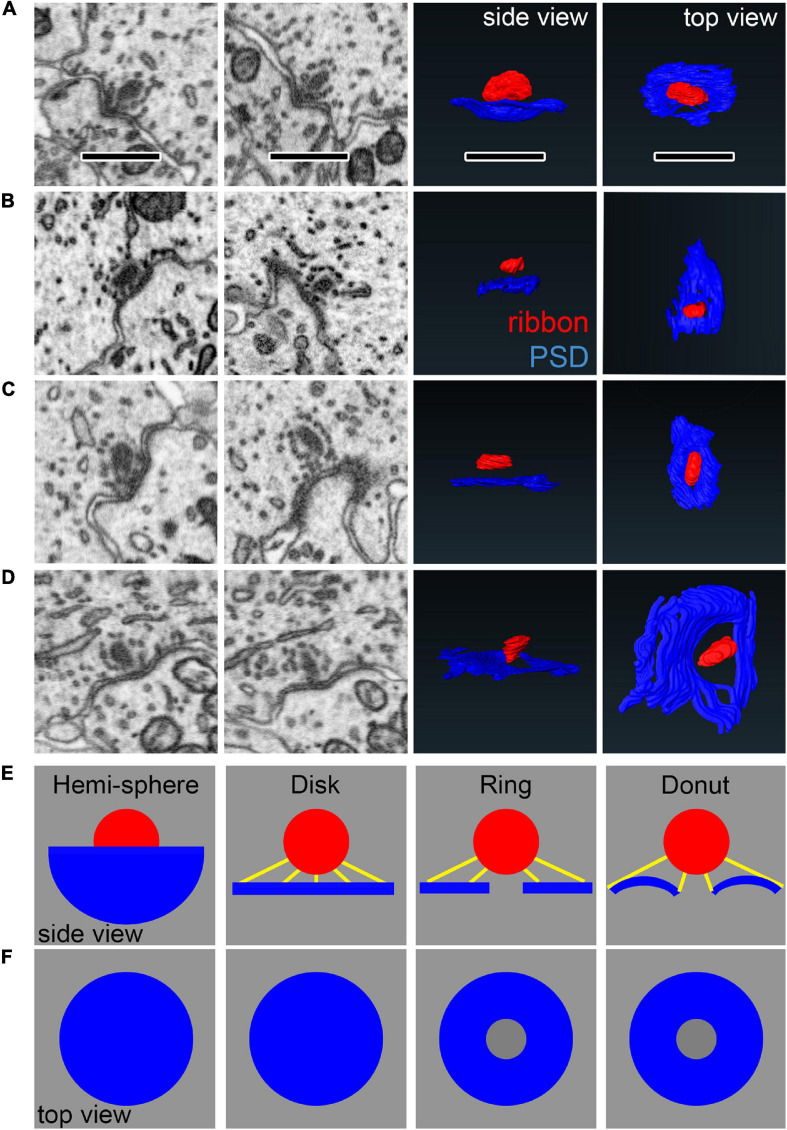
Comparing synapse morphology with models of synapse geometry. **(A–D)** Left: Observations of diversity in PSD shape in the raw data. Each row is one synapse shown from different perspectives. Right: Reconstructions in side views and top views. **(E–F)** Geometric models used for comparison with PSD shape, shown from the side **(E)** and top views **(F)**. Yellow lines show measurements of shortest distance from points on the PSD to the ribbon. Scale bars are approximately 1 μm.

When viewing ribbon synapse ultrastructure, we observed a variety of membrane curvatures at the PD/PSD apposition near the ribbon. The membrane curvature is often concave around the ribbon, then becoming more convex with further distance from the ribbon. However, the shape of the curvature of the synaptic cleft was complex, ranging from extremely convex or concave to relatively flat at the same synapse, depending on the section and the orientation, making them difficult to classify (Figures 10A–D). We therefore created 4 virtual synapses to span the range of shapes observed within and across synapses: the hemi-sphere, the disk, the ring, and the donut (Figures 10E,F). In each of the 4 models, the ribbon was a sphere with diameter of 300 nm. The edge of the ribbon was positioned 84 nm (12 voxels) away from the center of the PSD. The PSDs were constrained to have the same diameter (diameter of 3D projection on the 2D plane = 1400 nm). The *hemi-sphere* model has extreme concave curvature, in which the ribbon sits inside the “bowl” shape of the PSD. The *disk* model is flat, having no curvature. The *ring* is the disk with a hole in the center of 250 nm diameter. The *donut* includes the 250 nm diameter hole in the center, then has a concave curvature near the ribbon, becoming convex with further distance. Below, we will compare the shapes of these models with the shapes of ribbon synapses.

**FIGURE 11:**
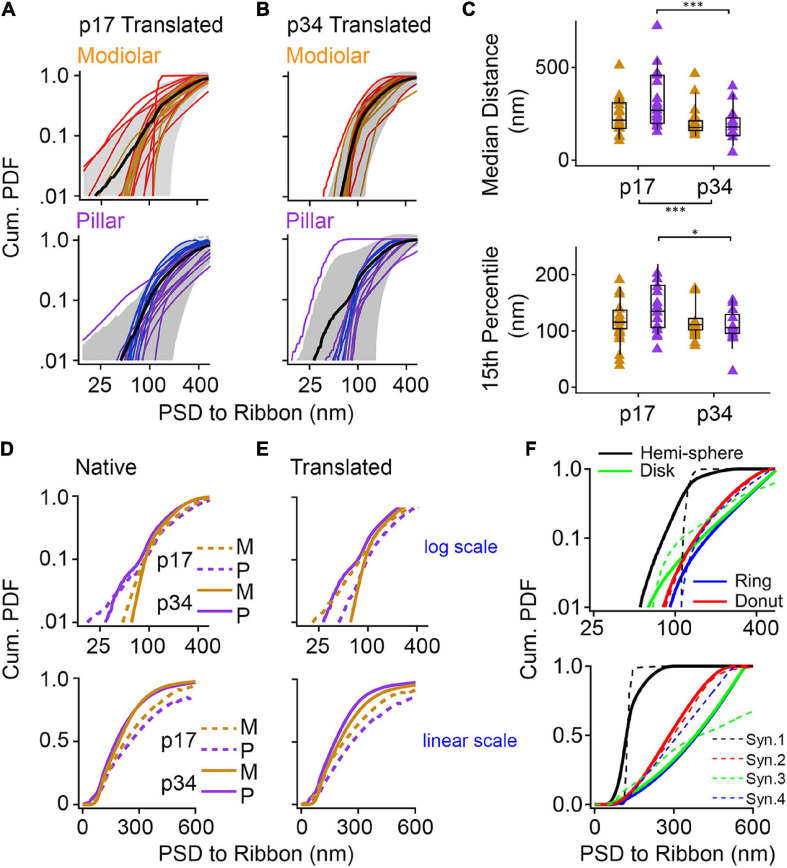
Post-synaptic density (PSD) shape measurements and comparison with geometric models. **(A,B)** Proximity of the ribbon and the synaptic cleft was quantified with shortest-distance measurements from points on the PSD to the synaptic ribbon, expressed as cumulative probability density functions (Cum. PDF). Synapses were grouped as modiolar (top) or pillar (bottom), according to the translated view, for p17 **(A)** and p34 **(B)**. The red lines in panels **(A,B)** denote synapses assigned as pillar in the native view; the blue lines denote synapses assigned as modiolar in the native view. **(C)** Box plots of the median distance (top) and 15% percentile distance (bottom) from PSD to Ribbon at p17 and p34. Gold is modiolar; purple is pillar. Significant shortening of median distances between p17 and p34 (*p* = 2.8E-03) came mainly from the pillar side (2.6E-03) which was also significant for the 15th percentile distance (4.7E-02). **(D,E)** Average cumulative probability density functions of each group in the native **(D)** and translated **(E)** views. Top graphs are on a log scale and bottom graphs are on a linear scale. **(F)** Cumulative probability density functions of the four model synapses from Figure 10 (solid lines). Dashed lines are data from synapses depicted in Figure 10: Black dashed line is 10A; Green dashed line is 10B; Blue dashed line is 10C; Red dashed line is 10D. *p < 0.05, ***p < 0.001.

We developed a novel, objective measurement of synapse shape and size. For the ribbon-facing surface of each segmented PSD, we measured the shortest distance from each vertex of the segmented model to the surface of the synaptic ribbon (Figure 10E). From the list of distance measurements for each synapse, we generated a histogram. Displayed as normalized cumulative histograms or probability density functions (Cum. PDF) for each synapse, each curve shows the distances measured from the PSD to the surface of the ribbon on the X-axis (Figure 11A: p17 modiolar (upper), p17 pillar (lower); Figure 11B: p34 modiolar (upper), p34 pillar (lower). For group comparisons, we extracted the median distance for each synapse and the 15th percentile distance for each synapse (Figure 11C). From p17 to p34, the range of median distances per synapse was significantly reduced from 103 – 723 nm to 42 – 466 nm (Table 1, median of medians reduced from 259 to 191 nm, *p* = 2.8e^–3^). In both the native and translated views, this developmental reduction in median distance was significant only on the pillar side (*p* = 1.1e^–2^, native; *p* = 2.6e^–3^, translated, Figure 11C). However, there was no significant modiolar-pillar difference at either age in either view (Figure 11C, Table 1 and Supplementary Table 1). Unlike the medians, the 15^*th*^ percentile distances were not significantly changed overall from p17 to p34, however, like the medians, there was a developmental reduction specifically on the pillar side (Figure 11C, lower). Therefore, like the reduction in PSD SA and short axes, the distances between PSDs and ribbons is reduced from p17 to p34. However, unlike the reduction in PSD SA and short axes on the modiolar side in the translated view, the maturational reduction in PSD-ribbon distance was found on the pillar side, suggesting that these measurements captured a different aspect of morphological maturation. For each of the four groups (p17 M, p17 P, p34 M, p34 P) we calculated the mean Cum. PDF for the native view (Figure 11D) and translated view (Figure 11E) displayed on a log-log scale (upper) or linear-linear scale (lower). Developmental effects (p17 vs. p34) were larger than spatial effects (M vs. P), and the largest developmental effect was on the pillar side, as is most clearly seen for the longest distances on the linear scale in the translated view (Figure 11E, lower).

To model synapse shape with this approach, we made these PDF measurements also on the four virtual synapses created from geometric shapes in Figures 10E,F. These PDFs are displayed alongside the group means on both the log scale and the linear scale (Figure 11F, solid lines). Overlaid in dashed lines are PDFs from four synapses resembling each of the four models represented by different colors. The PDF of the hemi-sphere has distances constrained to a very narrow range, but not identical distances as would be the case if the ribbon and the hemi-sphere had identical centers (Figure 11F, black line). The PDFs of the disk and the ring are very similar, particularly on a linear scale (Figure 11F, green and blue lines). On a log scale, they are seen to diverge at the low end because the ring is missing the shortest distances that are present on the disk (Figure 11F, upper). The PDF of the donut begins near that of the ring, due to the hole, but then rises more slowly like the hemi-sphere due to membrane curvature allowing for more short distances. The shapes of the PDFs for the real synapses appeared to most closely resemble the models that included curvature – the hemi-sphere and the donut. The PDFs of the flat models (disk and ring) had a feature not seen in the data – an ever-increasing slope – most clearly visible when displayed on the linear scale (Figure 11F, lower). Upon inspection of the pillar-side PDFs at p17 and p34 (Figures 11A,B, lower) the most obvious difference is a reduction in synapses with shapes that most closely resemble the disk and the ring models on the log scale (Figure 11F, upper, blue and green lines). This may reflect emergence of membrane curvature and/or a shift toward more compact synapses as they mature on the pillar side, possibly toward tighter spatial coupling.

### Modiolar/Pillar Difference in Ribbon-Associated Vesicles Emerges With Ribbon Gradient as Membrane-Associated Vesicles Are Reduced

**FIGURE 12:**
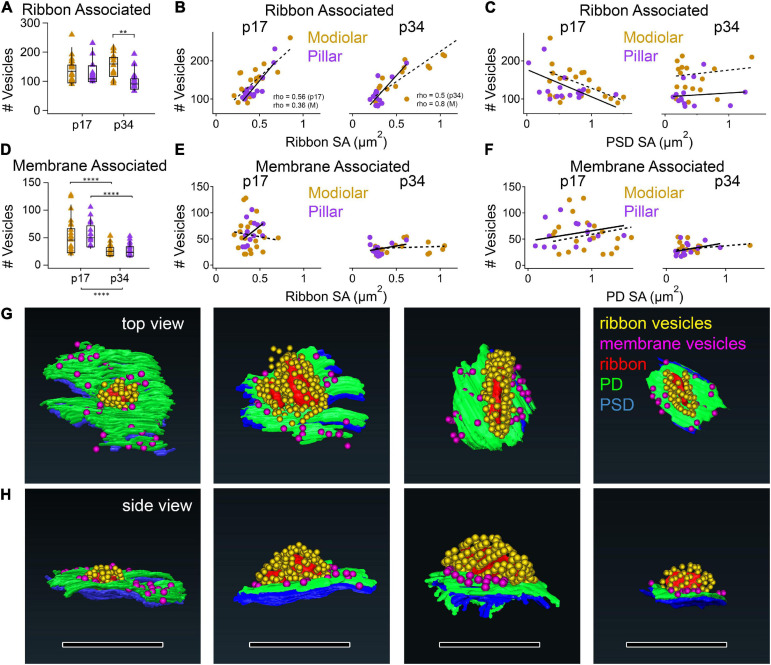
Vesicle content vs. synapse size by M-P position at p17 and p34. **(A)** Box plots of ribbon associated vesicles. **(B,C)** p17 (left) and p34 (right) ribbon-associated synaptic vesicles per synapse vs. Ribbon SA (B) or PSD SA (C) for modiolar (gold) or pillar synapses (purple). The correlations between #Vesicles/synapse and Ribbon SA were significant (p17 Pillar, *p* = 3.0E-04; Modiolar, *p* = 3.1E-02; p34 Pillar, *p* = 1.7E-09; Modiolar, *p* = 3.4E-04). **(D)** Box plots of membrane-associated vesicles. **(E,F)** p17 (left) and p34 (right) membrane-associated synaptic vesicles per synapse vs. ribbon SA **(E)** and PD SA **(F)**. **(G,H)** Reconstructions of synapses in the top **(G)** and side views **(H)**. Ribbon is red; PD is green; PSD is blue; Ribbon-associated vesicles are yellow; Membrane-associated vesicles are magenta. Scale bars are approximately 1 μm. **p < 0.01, ****p < 0.0001.

The numbers of synaptic vesicles within 80 nm of the surface of the ribbon (i.e., the number of ribbon-associated vesicles) did not change significantly from p17 to p34, either overall or on the modiolar or pillar sides in either view (Table 1 and Supplementary Table 1, Supplementary Figures 3A,B). Neither was there a significant habenular-cuticular gradient (Supplementary Figure 3C,D). However, in the translated view at p34 only, we found significantly more ribbon-associated vesicles at synapses on the modiolar side than the pillar side (Figure 12A, 171 ± 59 vs. 102 ± 34 vesicles per ribbon; *p* = 1.4e^–3^; Table 1), and a significant modiolar-pillar gradient (Supplementary Figure 3B, right side, Spearman’s rho = −0.55, *p* = 1.3e^–3^). We found no such differences or spatial gradients along the habenular-cuticular axis (Supplementary Figure 3C,D). There was a strong positive correlation between ribbon-associated vesicles and ribbon SA at p17 (Spearman’s rho = 0.56) and p34 (Spearman’s rho = 0.50; Figure 12B). When splitting synapses between modiolar and pillar, only the modiolar side correlations were significant (Figure 12B, Spearman’s rho = 0.36 at p17 and 0.8 at p34). As a function of PSD or PD SA, no significant correlation was found with ribbon-associated vesicles (Figure 12C).

The numbers of synaptic vesicles within 20 nm of the PD were counted as the membrane-associated pool, which was reduced significantly from p17 to p34 (Figure 12D, *p* = 2.1e^–6^) with changes occurring predominantly on the pillar side in the native view and on both the modiolar and pillar side in the translated view (Table 1 and Supplementary Table 1). The range of membrane-associated vesicles was larger at p17 (21 – 128 vesicles/synapse) than p34 (17 – 54 vesicles/synapse). No M-P or H-C gradients or group difference was detected, except for p17 in the native view, where the pillar side synapses had more vesicles than those on the modiolar side (Supplementary Figure 4A). There was a positive slope in the relationship between membrane-associated vesicles and ribbon SA on the pillar side and a negative slope on the modiolar side at p17 (Figure 12E), but these correlations were not significant. There was a slight positive slope in the relationship between membrane-associated vesicles and ribbon SA at p34, and with PD SA at p17 and p34, but none were significant with or without a few double-ribbon outliers removed (Figures 12E,F).

In conclusion, the large developmental reduction in the pool of membrane-associated vesicles was not seen in the ribbon-associated pool. The significant positive correlation between ribbon SA and ribbon-associated vesicles was not seen between ribbon SA and membrane-associated vesicles, nor between PD or PSD SA and ribbon-associated vesicles. Neither was there a significant correlation between PD or PSD SA and membrane-associated vesicles, although it was slightly positive as expected if the packing density of docked vesicles is similar at PDs of different sizes.

## Discussion

These observations of late postnatal development characterize the properties of mature synapses by comparing samples from the ears of mice at two ages: p17, just after hearing onset, and p34, when the cochlea is fully developed. Studies underway will compare synaptic anatomy with afferent and efferent fiber morphologies from the same data set. Subsequent studies should address morphological synaptic plasticity with manipulation of auditory inputs, as well as relate synaptic and afferent fiber morphologies to the physiological response properties of ANFs, as has been done in the cat (Merchan-Perez and Liberman, 1996).

Cochlear ribbon synapses are glutamatergic, like most excitatory chemical synapses in the brain. Synaptic membrane densities in the cochlea are much larger than those in the brain. In the rat visual cortex, glutamatergic synapses are approximately 300 nm in diameter with a surface area of ∼0.1 μm^2^, on average (Aoki et al., 2001). In the rat somatosensory cortex, they are approximately 0.05 – 0.07 μm^2^ (Santuy et al., 2018). For cochlear ribbon synapses, PSD surface area from 2D measurements in the cat ranged 0.2 – 0.5 μm^2^ (Liberman, 1980; Merchan-Perez and Liberman, 1996), and in the mouse PSD length ranged up to ∼1500 nm (Michanski et al., 2019). Consistent with this, in the mouse cochlea we found median PSD surface areas of 0.58 μm^2^ at p17 and 0.31 μm^2^ at p34, without any correction for tissue shrinkage. Large synaptic membrane densities may be important for supporting high rates and/or amplitudes of synaptic transmission. Although afferent PSDs of the cochlea are generally large, we observed a considerable 10-fold range from 0.13 to 1.36 μm^2^. Assuming a constant packing density of 900 AMPA-type glutamate receptors per μm^2^ (Momiyama et al., 2003), these cochlear synapses would contain between 117 and 1224 receptors per afferent terminal, whereas a typical central synapse of 0.1 μm^2^ would have only ∼90 receptors.

### Coordinated Maturation of Pre- and Post-Synaptic Membrane Densities After Hearing Onset

Here, we focused on comparisons of pre- and post-synaptic morphologies during cochlear maturation after hearing onset, by reconstruction of all afferent synapses in the acquired FIB-SEM volume, with attention to synapse position on the IHC. We observed large differences in cochlear synapse morphology between p17 and p34 including reduction in PD and PSD size, and a change in the distribution of ribbon sizes from unimodal to bimodal (Table 1 and Figure 4). The size and shape of the PD was very similar to that of the PSD at individual paired synapses, despite the large range of sizes at both ages, suggesting that paired PDs and PSDs changed in a coordinated fashion as they became smaller and less rounded, overall. This trans-synaptic coordination of membrane density morphology does not appear to extend to the ribbon. We did not see a correlation of ribbon morphology with pre- or post-synaptic morphology (Figures 4G–J, 9C,D). This suggests that different signals may influence developmental changes in synapse morphology between the local environment of the afferent terminal in the tissue and the cytoplasmic environment of the IHC ribbon.

Cochlear synaptic maturation in mice continues beyond the 2nd postnatal week while synapse number remains unchanged. Studies of early postnatal development through the onset of hearing function in the second and third postnatal weeks have assessed ultrastructural changes at the synapse with EM and assessed molecular anatomical changes using fluorescence with antibodies to presynaptic voltage-gated Ca^2+^ channels (Ca_*V*_1.3) and postsynaptic AMPARs (Meyer et al., 2009; Frank et al., 2010; Wong et al., 2014; Neef et al., 2018). These studies demonstrated that Ca_*V*_1.3 channels are located throughout the basolateral membrane of the IHC in the first postnatal week. Around the end of the second postnatal week, these channels become confined to stripe-like arrays at active zones where ribbons are located. At the same time, ribbons become fewer in number and larger in size, and AMPARs consolidate to form smaller, denser plaques that often form a ring-like structure with a void in the center. In the present study, we observed consolidation of the PSD from p17 to p34 with shortening of the short axis. The long axis of the PSD remained relatively constant from p17 to p34, and the length of the long axis on average (∼ 1.1 μm) was very similar to the length of the AMPAR cluster measured with STED microscopy. On the presynaptic side, the PD short axis shortened from ∼ 0.66 to 0.36 μm, on average, from p17 to p34. In comparison, Ca_*V*_1.3 immuno-labeled clusters have long and short axes of approximately 0.25 and 0.075 μm, on average, at p15-p18. We conclude that consolidation of membrane densities, as seen in other studies during early postnatal development, continued throughout late postnatal development after the onset of hearing function. Taken together, these studies suggest that Ca_*V*_1.3 channels occupy a more restricted region in the PD relative to the occupancy of AMPARs in the PSD.

### Synapse Position on the IHC, and IHC Position in the Organ of Corti

Traditionally, in electron microscopy the modiolar-side (M) and pillar-side (P) synapses have been identified, one IHC at a time, based on which hemisphere the synapse resides. More recently with confocal microscopy of cochlear whole-mounts, this is done by rotating the image volume 90 degrees for projection onto the ZY plane, to view a row of ∼10 IHCs superimposed on each other in a row, and then applying a user-defined line on the ZY plane to best capture the differences in ribbon size between two groups of synapses: one positioned on the modiolar and/or basal side of the line; and the other positioned on the pillar and/or apical side of the line (Liberman et al., 2011, see their Figure 4b; Liberman and Liberman, 2015). This method includes any spatial differences due to relative position of adjacent IHC basolateral membranes along the M-P axis in the tissue, which can obscure cell-centric M-P trends. Modiolar and pillar position has also been assigned by superimposing synapses from each IHC individually onto a common central axis (as if the IHC were a cylinder) for group analysis, using the IHC central axis on the ZY plane to define an orthogonal plane (XY) dividing modiolar-side from pillar-side synapses along the M-P (Z) axis (e.g., Ohn et al., 2016). The latter method seeks to disambiguate IHC position in tissue from synapse position on the IHC.

In this study, we determined modiolar-side and pillar-side synapses using both methods, referring to them as the native view and the translated view. Synapses can change modiolar or pillar groups depending on the view because synapses on the modiolar face of one IHC can occupy the same M-P position in the tissue as synapses on the pillar face of an adjacent IHC (Figure 6). For p17: 4 synapses (6 ribbons) assigned M in the native view became P in the translated view; and 12 synapses assigned P in the native view became M in the translated view. For p34: 6 synapses assigned M in the native view became P in the translated view; 7 synapses (8 ribbons) assigned P in the native view became M in the translated view.

We found a significant M-P gradient in ribbon size only at p34 in the translated view (Figure 7), demonstrating that cell-centric M-P differences can be obfuscated by relative positions of the cells in the tissue. With a larger sample size, it is likely we would have detected significant M-P gradients in the native view, as well. This M-P gradient in ribbon size is consistent with trends observed in EM of the adult cat (Liberman, 1980; Kantardzhieva et al., 2013) and in the developing postnatal mouse from p9 to p34, although M vs. P comparisons in previous studies are often not significantly different (Michanski et al., 2019), perhaps due to obfuscation by cell position in the tissue. Specifically, Kantardzhieva et al. (2013) reported average ribbon volumes of 3.1 vs. 3.3 nm^3^ × 10^6^ for the two groups of synapses deemed hi- or low-spontaneous-rate, for a difference of only ∼4% in adult cat. Michanski et al. (2019) reported average ribbon volumes of ∼8 vs. ∼6.5 nm^3^ × 10^6^ in modiolar vs. pillar groups, for a difference of ∼18% in the p34 mouse. Here, we found much larger differences, as much as 100%: average ribbon volumes were 4.9 and 2.4 nm^3^ × 10^6^ in modiolar and pillar groups, respectively, in the p34 mouse after translating synapse position. Before translation, differences were smaller: 3.9 vs. 3.0 nm^3^ × 10^6^.

Confocal analysis in mouse has used immunohisto- fluorescence to demonstrate spatial gradients of ribbon size (using anti-CtBP2) and post-synaptic density size (using anti-GluA2). Such gradients in the sizes of synaptic puncta may depend on when during development, where in the cochlea, and what strain of mouse one is observing. For example, ribbon volumes were greater on the pillar side at p14, then greater on the modiolar side at p28 in most cochlear regions, while postsynaptic volumes were greater on the pillar side at p14 and at p28 in most cochlear regions of CBA/CaJ mice (Liberman and Liberman, 2016). The size of M-P differences in ribbon volume and the existence of M-P differences in GluA2 volume seem to depend on the rodent species or mouse strain. For example, although CBA/CaJ mice have opposing gradients of CtBP2 and GluA2 volume, in comparison the C57BL/6 mice, FVB/NJ mice, and gerbils have concurrent gradients (Liberman et al., 2011; Zhang et al., 2018; Reijntjes et al., 2020).

### Ribbon Long Axes Elongate on the Modiolar Side With Maturation After Hearing Onset

Our analysis utilizes the 7 nm isotropic voxels from FIB-SEM which allowed us to completely reconstruct the ribbon synapses without distortion and to obtain the longest and shortest axis of each ribbon from its 3D model (Figures 2–4). We determined that ribbon long axes grow over the course of development, becoming less rounded, predominately on the modiolar side of the IHCs (Figures 7, 9). Consistent with this, previous studies of serial EM on adult mid-cochlear mouse synapses found columnar ribbons on the modiolar side and ovular ribbons on the pillar side (Stamataki et al., 2006). In studies of ribbon size in the mouse from EM ultrathin sections, 2D measures of ribbon axes (the height and width) of mature ribbons ranged ∼50 – 400 nm (Michanski et al., 2019). Our observed ranges (p17: 263 – 478 nm; p34: 271 – 813 nm) are larger than those reported previously in the adult mouse and cat (Kantardzhieva et al., 2013), presumably because we used a 3D reconstruction to measure the longest axes, which should provide more realistic estimates from ribbon long-dimensions that are difficult to capture without serial reconstruction. Reported short axis measurement for ribbons at p17 were ∼120 nm, on average, using 2D measures (Meyer et al., 2009). Our average short axis measures (p17, 178 nm; p34, 159 nm) are larger than those reported previously, presumably because our short axis was constrained to intersect with the center of mass of the entire 3D object.

Previous work using schematic reconstruction of serial micrographs of the adult cat reported low SR fibers synapsing on the modiolar side and having greater ribbon long-axes than ribbons of medium or high SR fibers synapsing on the pillar side (Merchan-Perez and Liberman, 1996). Our finding of longer ribbon long axes on the modiolar-side suggests that modiolar-side synapses may correspond to low-SR fibers also in the mouse. However, the localization of low-SR and high-SR fibers around the circumference of the IHC in mouse still needs to be investigated. Interestingly, there may be important differences between mice and cats in this regard. Recently, studies of the physiology of ANF’s in mouse following noise exposure suggested that the population fraction of low-SR and high-SR fibers did not change after noise (Suthakar and Liberman, 2020; abstract #73 of the 2020 Midwinter meeting of the Association for Research in Otolaryngology). This would be different from observations in guinea pig, where noise exposure selectively reduced the population of low SR ANFs (Furman et al., 2013). In the gerbil, there are fewer low-SR fibers with age (Schmiedt et al., 1996). More work is needed to understand if mice and men follow this pattern of low-SR ANF vulnerability to noise and aging.

### PD and PSD Short Axes Shorten on the Modiolar Side With Maturation After Hearing Onset

Interestingly, PD and PSD short axes narrow over development from p17 to p34 predominantly on the modiolar side, becoming more like pillar-side PDs and PSD’s, while long axes remain unchanged (Figure 8). This may suggest the pillar side PSDs began to narrow already before p17, consistent with the birth order of the ANFs beginning with those that differentiate into type-Ia, which tend to synapse on the pillar side and are hypothesized to correspond with the hi-spontaneous rate/low-threshold fibers (Shrestha et al., 2018). Curiously, our PSD-ribbon proximity measurements (Figures 10, 11) revealed significant changes with maturation predominantly on the pillar side, which may reflect shape changes not captured by measures of surface area or axis length. The maturational changes to the PD and PSD were larger than M-P differences at either age (Figures 8, 11). We observed a range of PSD shapes on both the modiolar and pillar sides, without any obvious segregation, suggesting that overall synapse shapes are heterogeneous regardless of position of ANF terminal innervation onto midcochlear IHCs of the mouse. More work is needed to characterize synapse shape and membrane curvature, and to understand its relationship to fiber properties.

### Membrane-Associated Vesicle Pool Matures as a Modiolar/Pillar Difference in Ribbon-Associated Vesicles Emerges

The organization of synaptic vesicle pools around the ribbon and how vesicles are replenished during continuous stimulation of ribbon synapses is not well understood. There are three broad classes of synaptic vesicles: ribbon-associated vesicles that are tethered or near the ribbon, docked vesicles that are near the pre-synaptic density and can be released rapidly, and the recycling pool of vesicles that are ready to replenish the store of vesicles at the synapse (Chakrabarti and Wichmann, 2019). These morphological pools have physiological correlates measurable by membrane capacitance (for review, see Glowatzki et al., 2008). Most comparisons of morphological vesicle pools and their physiological correlates have focused on early postnatal development before and after the onset of hearing function around p14. Here we focused on later postnatal maturation of vesicle pools after the onset of hearing.

We found a significant maturational decrease in the number of membrane-associated vesicles from p17 to p34 (from 53 to 30 vesicles per synapse; Figure 12; Table 1) on both the modiolar and pillar sides. This reduction was similar in proportion to the decrease in PD surface area from p17 to p34 (0.75 to 0.33 μm^2^). There was no M-P difference in membrane-associated vesicles or surface area of the PD. Although there was significant heterogeneity in membrane-associated vesicle number per synapse at p17 and p34, we found no clear relationship between vesicle number and ribbon, PD, or PSD size within those groups. The physiological correlate of the maturational reduction in membrane associated vesicles is unknown. However, we note that in the rat cochlea, from p14 otoferlin expression increases to reach mature levels in the IHCs at p24 (Schug et al., 2006). The decrease in membrane-associated vesicles over development could be related to otoferlin’s role in calcium-mediated exocytosis. Perhaps more membrane-associated vesicles are present at p17 because otoferlin levels are lower. In general, if the molecular machinery is not yet fully in place to facilitate mature exocytosis or endocytosis, then vesicle turnover could be slower with vesicles spending longer times at the PD before exocytosis, and thus more are visible at p17 than p34.

We found no maturational decrease in the number of ribbon-associated vesicles (Figure 12). In contrast, in studies of moues IHCs from the apical cochlea, counting all the synaptic vesicles within 80 nm of the surface of the ribbon, the numbers of vesicles per synapse ranged ∼50 – 160 at p17 and ∼30 – 100 at p34, demonstrating a developmental decrease in the ribbon-associated pool (Michanski et al., 2019). We found somewhat larger numbers from IHCs of the mid-cochlea, where we found no developmental reduction in ribbon-associated vesicles. This difference may be related to the observation that mid-cochlear synapses have greater numbers of ribbon-associated vesicles compared to apical-cochlear synapses in p17 mice (Meyer et al., 2009). As the surface area of the ribbon increases, there is more space for ribbon-associated vesicles. As expected, we found a strong correlation between ribbon size and the ribbon-associated vesicle pool, as previously reported (Stamataki et al., 2006). Finally, at p34 we found greater numbers of ribbon-associate vesicles on the modiolar than the pillar side, consistent with the significant spatial difference in ribbon size. If, as in the cat, larger ribbons provide input to the low-spontaneous rate/high-threshold synapses, then we must understand how ribbons with more vesicles drive fibers at lower rates. One potential mechanism involves differences in voltage-dependence of Ca^2+^ current activation, whereby modiolar synapses activate only for larger stimuli (Ohn et al., 2016). Future work in mice should (1) determine the innervation pattern around the IHC circumference with respect to ANF spontaneous rate, (2) correlate ultrastructural properties of synapses and fibers, and (3) relate these observations to ANF subtype Ia, Ib, Ic molecular identity.

## Data Availability Statement

The original contributions presented in the study are included in the article/Supplementary Material, further inquiries can be directed to the corresponding author/s.

## Ethics Statement

The animal study was reviewed and approved by the Institutional Animal Care and Use Committee at Washington University in St. Louis (20180125).

## Author Contributions

MR designed the study. MR and JF acquired the funding. MR, SP, and MJ prepared the samples and collected the data. SP, HC, NS, AF, SG, KV, AS, MN, AB, GI, BD, JC, and HP annotated the raw data. SP developed unpublished analysis methods. SP, MJ, and MR analyzed the data. SP and MR wrote the manuscript. SP, MJ, and MR revised the manuscript. All authors contributed to the article and approved the submitted version.

## Conflict of Interest

MJ was employed by TESCAN. The remaining authors declare that the research was conducted in the absence of any commercial or financial relationships that could be construed as a potential conflict of interest.
