# Supplementary material for: Maturation of Heterogeneity in Afferent Synapse Ultrastructure in the Mouse Cochlea
Source: Front Synaptic Neurosci. 2021 Jun 17;13:678575. doi: 10.3389/fnsyn.2021.678575 (PMC8248813; doi:10.3389/fnsyn.2021.678575)
Supplement: Supplementary file 11 [file Data_Sheet_1.PDF]

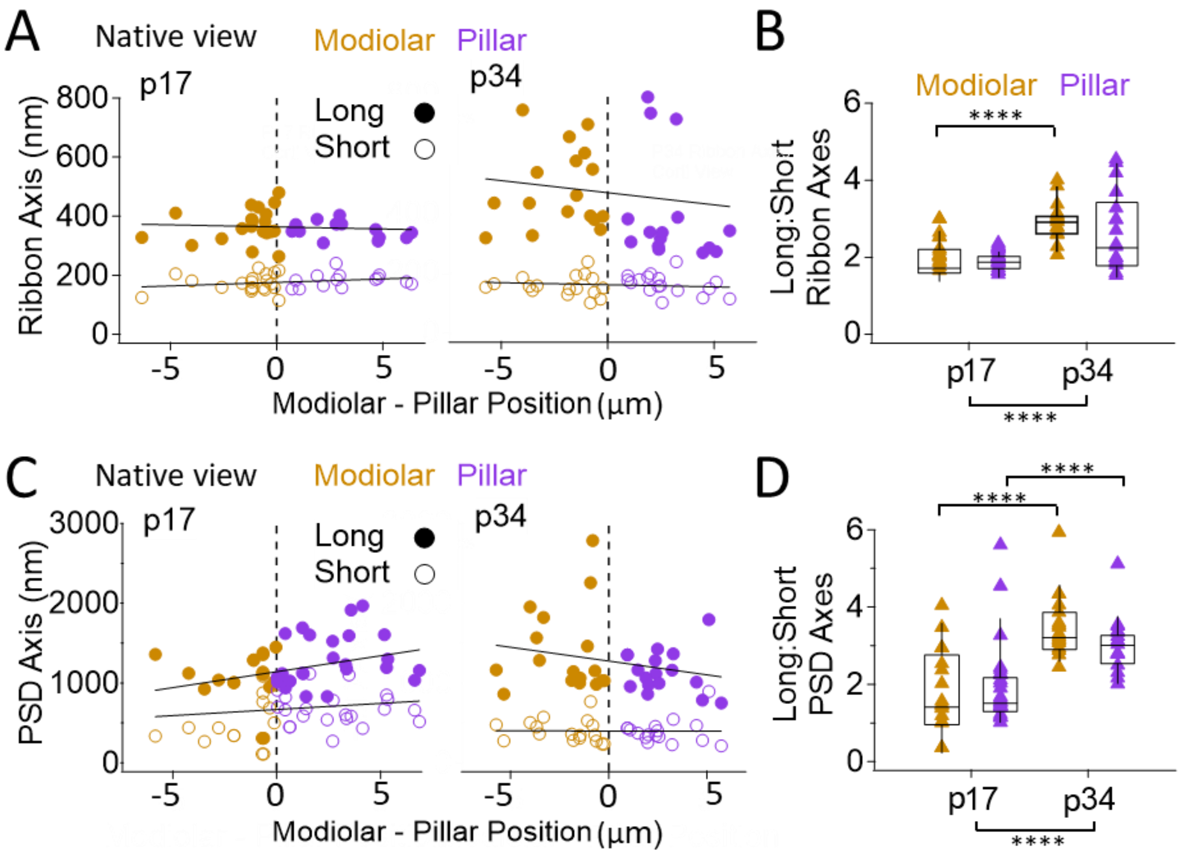

Figure S1. Ribbon and PSD long and short axes at p17 and p34 by synapse position in the tissue (i.e., native view). (A) Ribbon long and short axes versus synapse position along the modiolar-pillar axis for p17 (left) and p34 (right). **(B)** Long : short ribbon axis ratios at both ages. Modiolar (gold), Pillar (purple). **(C)** PSD long and short axes versus synapse position along the modiolar-pillar axis for p17 (left) and p34 (right). **(D)** Long : short PSD axis ratios at both ages in modiolar (gold) and pillar (purple) groups. Within each box, horizontal line denotes the median; box extends the interquartile range; vertical line denote the 10-90 percentile range.

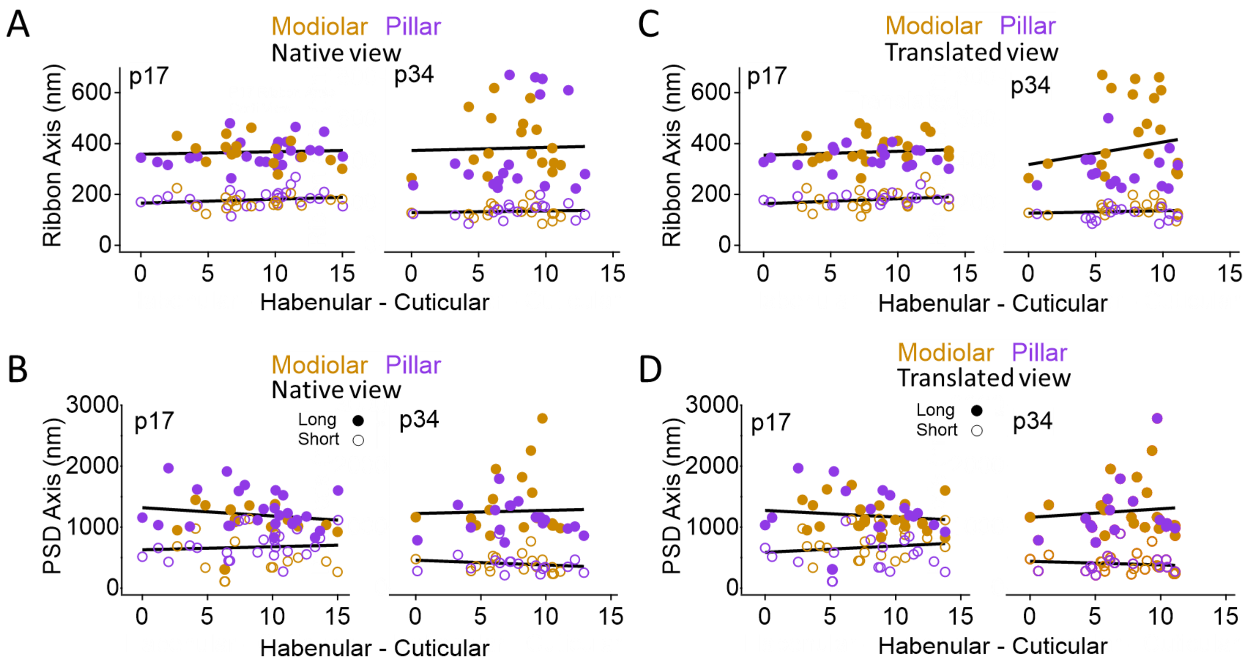

Figure S2. Habenular-Cuticular gradients of ribbon and PSD long and short axes at p17 and p34 in the native and translated views. (A) Ribbon long and short axes by synapse position along the length of the IHC from the basal pole to the apical pole (i.e., the habenular-cuticular axis) at p17 (left) and p34 (right) in the native view. Gold colored markers indicate modiolar-side synapses; purple-colored markers indicate pillar-side synapses. **(B)** As in panel A, for PSD long and short axes. **(C)** As in panel A, after aligning the basal-most synapse of each IHC (i.e., for synapse position in the translated view). **(D)** As in panel C, for PSD long and short axes.

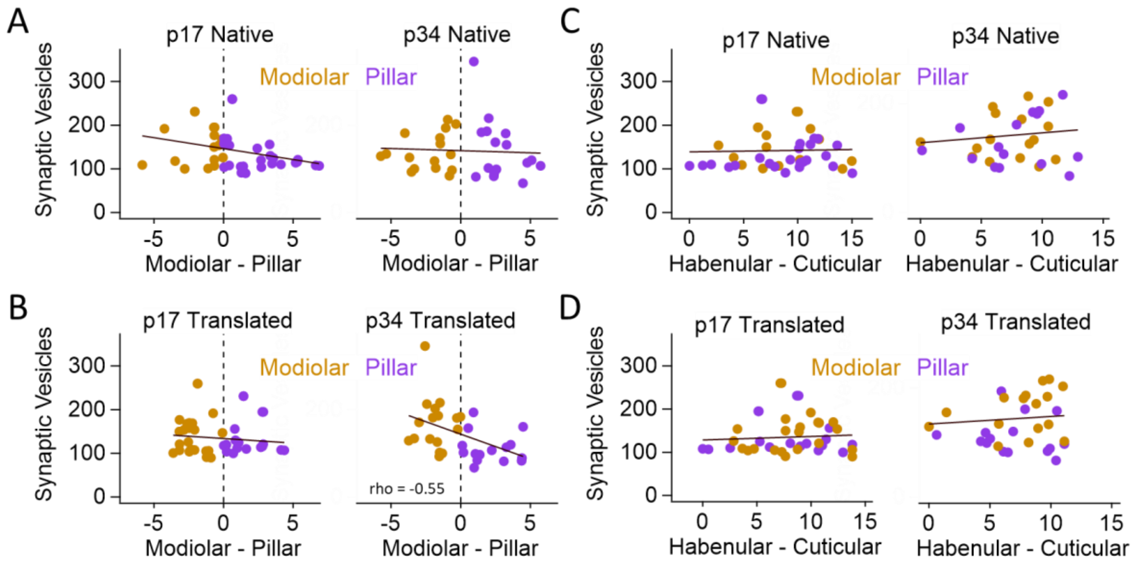

Figure S3. Ribbon-associated synaptic vesicles by synapse position along the modiolar-pillar and habenular-cuticular axes. (A) Number of ribbon-associated synaptic vesicles per synapse versus modiolar-pillar position for p17 (left) and p34 (right) in the native view. Gold colored markers indicate modiolar-side synapses; purple-colored markers indicate pillar-side synapses. **(B)** As in panel A, after IHC alignment along the M-P axis (i.e., translated view). (P34: p = 1.3E-03) **(C)** Vesicle number per synapse versus habenular-cuticular position at p17 (left) and p34 (right), native view. **(D)** As in panel C, for the translated view. Panels with rho listed have significant correlations.

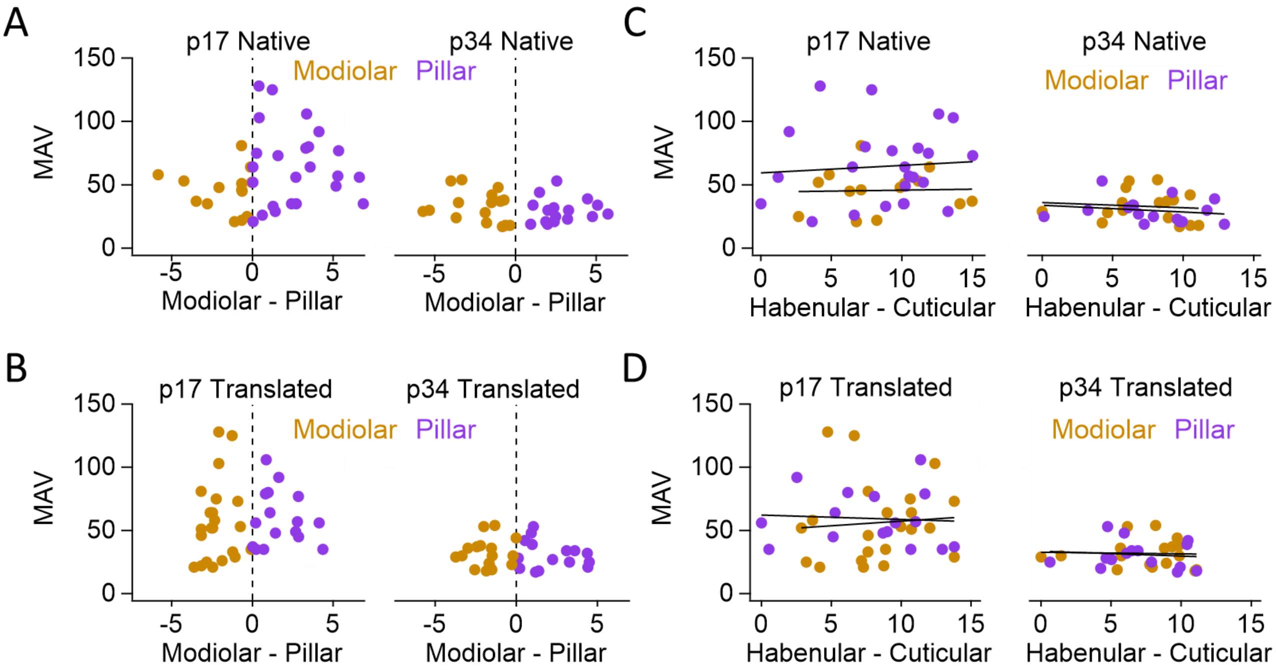

Figure S4. Membrane-associated vesicles by synapse position along the modiolar-pillar and habenular-cuticular axes. (A) Number of membrane-associated vesicles per synapse versus modiolar-pillar position for p17 (left) and p34 (right) in the native view. Gold colored markers indicate modiolar-side synapses; purple-colored markers indicate pillar-side synapses. **(B)** As in panel A, after IHC alignment along the M-P axis (i.e., translated view). **(C)** Vesicle number per synapse versus habenular-cuticular position at p17 (left) and p34 (right), native view. **(D)** As in panel C, for the translated view.

	Age	ALL (range)	Modiolar (range)	Pillar (range)	Modiolar vs. Pillar Wilcoxon p-value (effect size)
**Ribbon Volume**	P17	4.29 (0.80 - 10.93)	3.76 (2.29 - 9.53)	4.59 (0.80 - 10.93)	7.5E-02 (0.45)
	P34	3.29 (0.75 - 10.22)	3.94 (1.77 - 7.59)	2.99 (0.75 - 10.22)	5.3E-01 (0.37)
	p17 vs. p34 Wilcoxon p-value (effect size)	7.6E-02 (0.45)	8.4E-01 (0.10)	7.3E-02 (0.64)	
**Ribbon SA**	P17	0.34 (0.19 - 0.71)	0.35 (0.21 - 0.55)	0.39 (0.19 - 0.71)	7.7E-01 (0.37)
	P34	0.32 (0.20 - 1.07)	0.35 (0.24 - 1.05)	0.29 (0.20 - 1.07)	1.4E-01 (0.25)
	p17 vs. p34 Wilcoxon p-value (effect size)	5.9E-01 (0.20)	1.9E-01 (0.03)	1.1E-02 (0.46)	
**Ribbon Long Axis**	P17	357 (263 - 480)	372 (278 - 462)	350 (263 - 480)	6.5E-01 (0.45)
	P34	394 (271 - 813)	437 (321 - 751)	340 (271 - 813)	1.0 E-01 (0.56)
	p17 vs. p34 Wilcoxon p-value (effect size)	6.9E-02 (0.30)	6.6E-03 (0.68)	6.6E-01 (0.07)	
**Ribbon Short Axis**	P17	178 (144 - 269)	166 (124 - 225)	185 (114 - 269)	4.5E-02 (0.67)
	P34	159 (102 - 241)	155 (102 - 240)	170 (103 - 241)	5.3E-01 (0.46)
	p17 vs. p34 Wilcoxon p-value (effect size)	2.3E-02 (0.61)	2.9E-01 (0.38)	5.3E-02 (0.46)	
**Ribbon Long:Short Ratio**	P17	2.0 (1.6 - 3.0)	2.1 (1.7 - 3.0)	2.0 (1.6 - 2.4)	8.4E-02 (0.13)
	P34	3.0 (1.5 - 4.5)	3.0 (2.1 - 4.0)	2.3 (1.5 - 4.5)	1.6E-01 (0.85)
	p17 vs. p34 Wilcoxon p-value (effect size)	5.3E-06 (1.6)	3.6E-05 (2.1)	4.1E-02 (0.5)	
**PD SA**	P17	0.75 (0.11 - 1.62)	0.42 (0.11 - 1.41)	0.79 (0.33 - 1.62)	4.8E-02 (1.01)
	P34	0.33 (0.16 - 1.41)	0.34 (0.16 - 1.41)	0.33 (0.16 - 0.91)	8.6E-01 (0.02)
	p17 vs. p34 Wilcoxon p-value (effect size)	6.7E-06 (1.29)	1.3E-01 (0.23)	7.9E-06 (1.58)	
**PD Long Axis**	P17	1.12 (0.66 - 2.28)	1.15 (0.66 - 1.54)	1.24 (0.84 - 2.28)	1.1E-01 (0.31)
	P34	1.13 (0.82 - 2.3)	1.11 (0.91 - 2.31)	1.18 (0.82 - 1.80)	9.4E-01 (0.25)
	p17 vs. p34 Wilcoxon p-value (effect size)	2.2E-01 (0.17)	7.6E-01 (0.15)	1.7E-01 (0.19)	
**PD Short Axis**	P17	0.75 (0.22 - 1.29)	0.48 (0.22 - 1.29)	0.80 (0.28 - 1.16)	1.6E-01 (1.09)
	P34	0.38 (0.22 - 0.78)	0.39 (0.22 - 0.78)	0.37 (0.24 - 0.64)	7.8E-01 (0.15)
	p17 vs. p34 Wilcoxon p-value (effect size)	2.8E-07 (1.57)	8.5E-02 (0.36)	2.6E-07 (2.21)	
**PD Long:Short Ratio**	P17	1.6 (1.1 - 5.4)	2.3 (1.1 - 4.1)	1.5 (1.1 - 5.4)	5.5E-01 (0.72)
	P34	3.0 (1.9 - 4.9)	3.0 (2.5 - 4.4)	2.9 (1.9 - 4.9)	7.0E-01 (0.17)
	p17 vs. p34 Wilcoxon p-value (effect size)	7.0E-07 (1.56)	8.4E-03 (0.81)	2.6E-05 (1.52)	
**PSD SA**	P17	0.58 (0.03 - 1.50)	0.40 (0.03 - 1.30)	0.67 (0.32 - 1.50)	1.1E-01 (0.77)
	P34	0.31 (0.13 - 1.36)	0.31 (0.19 - 1.36)	0.31 (0.13 - 1.26)	5.8E-01 (0.01)
	p17 vs. p34 Wilcoxon p-value (effect size)	6.5E-04 (0.80)	5.0E-01 (0.22)	2.6E-05 (1.22)	
**PSD Long Axis**	P17	1.12 (0.31 - 1.97)	1.07 (0.11 - 1.45)	1.18 (0.83 - 1.97)	8.9E-02 (0.31)
	P34	1.15 (0.75 - 2.78)	1.15 (0.86 - 2.78)	1.20 (0.75 - 1.79)	2.0E-01 (0.08)
	p17 vs. p34 Wilcoxon p-value (effect size)	9.7E-01 (0.06)	1.2E-01 (0.18)	2.2E-01 (0.20)	
**PSD Short Axis**	P17	0.66 (0.11 - 1.28)	0.47 (0.27 - 1.28)	0.67 (0.27 - 1.13)	2.2E-01 (0.73)
	P34	0.36 (0.21 - 0.89)	0.36 (0.24 - 0.77)	0.37 (0.21 - 0.89)	8.3E-01 (0.09)
	p17 vs. p34 Wilcoxon p-value (effect size)	7.9E-06 (1.27)	1.0E-01 (0.45)	3.5E-06 (1.48)	
**PSD Long:Short Ratio**	P17	1.7 (1.0 - 5.6)	2.2 (1.0 - 4.0)	1.6 (5.6 - 1.0)	4.5E-01 (0.57)
	P34	3.2 (2.0 - 5.9)	3.3 (2.4 - 5.9)	3.1 (2.0 - 5.1)	5.6E-02 (0.27)
	p17 vs. p34 Wilcoxon p-value (effect size)	8.8E-09 (1.63)	7.8E-05 (1.32)	3.0E-05 (1.61)	
**PSD Shape Assesment (Medians)**	P17	259 (103 - 723)	261 (153 - 469)	245 (103 - 723)	7.7E-01 (0.12)
	P34	191 (42 - 466)	202 (147 - 466)	177 (41 - 373)	1.4E-01 (0.32)
	p17 vs. p34 Wilcoxon p-value (effect size)	2.8E-03 (0.63)	1.9E-01 (0.72)	1.1E-02 (0.56)	
**PSD Shape Assesment (15th Percentile)**	P17	109 (38 - 264)	102 (90 - 170)	123 (38 - 264)	1.6E-01 (0.49)
	P34	102 (29 - 176)	102 (79 - 173)	102 (29 - 176)	8.3E-01 (0.0)
	p17 vs. p34 Wilcoxon p-value (effect size)	9.3E-02 (0.19)	6.1E-01 (0.0)	1.7E-01 (0.46)	
**Ribbon Associated Vesicles**	P17	120 (90 - 260)	138 (100 - 231)	115 (90 - 260)	1.8E-01 (0.62)
	P34	128 (67 - 346)	131 (84 - 213)	117 (67 - 346)	6.5E-01 (0.25)
	p17 vs. p34 Wilcoxon p-value (effect size)	1.0 (0.15)	7.0E-01 (0.17)	9.9E-01 (0.04)	
**Membrane Associated Vesicles**	P17	53 (21 - 128)	47 (21 - 81)	61 (21 - 128)	5.9E-02 (0.53)
	P34	30 (17 - 54)	33 (17 - 54)	29 (19 - 53)	5.4E-01 (0.43)
	p17 vs. p34 Wilcoxon p-value (effect size)	2.1E-06 (1.06)	3.9E-02 (1.00)	1.6E-05 (1.34)	

Table S1. Median Ribbon volume ($nm^3 \times 10^6$), Ribbon and PSD SA (μm^2), Ribbon and PSD Axes (nm), RCPH (nm), and Vesicles per synapse at p17 and p34 by Modiolar-Pillar position in the native view. For p17: N = 41 (23M/18P); p34: N = 34 (19M/15P).

Movie S1. p17 synapse 32. This 40s movie goes through one synapse beginning in the afferent terminal, proceeding through the synaptic cleft for a direct face view of the synaptic ribbon, ending in the hair cell cytoplasm among synaptic vesicles, endosomes, cisterns, and mitochondria.

Movie S2. p17 synapse 33. This 40s movie goes through one synapse from the side, beginning inside the afferent fiber with the hair cell emerging on the left and the afferent terminal on the right.

Movie S3. p17 synapse 37. This 40s movie begins in the hair cell with the afferent fiber emerging on the left to contact a double ribbon synapse on the right, ending with several efferent contacts onto the postsynaptic afferent fiber terminal near the afferent ribbon synapse.

Movie S4. Front view - each 50th image acquired of p17, downsampled 2x2. This perspective shows the front view, as the images were collected. This 10s movie begins with the inner boarder cells before passing through the hair cells into the pillar cells, ending with the tunnel of Corti, on right.

Movie S5. Radial view - virtual re-slice of p17, each 100th image, downsampled 2x2. This is the side-view, or radial perspective (like a mid-modiolar section), generated from virtual sections of the digital images. This 30s movie runs along the tonotopic dimension of the organ of Corti, with modiolar side on the left and pillar side on the right.

Movie S6. Top-down view - virtual reslice of p17, each 30th image, downsampled 2x2. This is the top-down perspective, generated from virtual sections of the digital images. This 15s movie runs from below the hair cell nucleus toward the basal pole of the hair cell and then the habenula perforate. Modiolar side is on the left and pillar side on the right.

Movie S7. p34 Cell2 - synaptic cluster on pillar face of IHC. This 10s movie includes 4 synapses far apart.

Movie S8. p34 Cell3 - synaptic cluster on modiolar base of IHC. This 40s movie includes 4 synapses closely spaced.

Movie S9. p17 central hair cell model with synapses. This 20s movie begins with the segmented central IHC in white with segmented synapses in red (ribbons), green (PDs), and blue (PSDs). The data scrolls through the model from the perspective from which the images were acquired, with the habenular-cuticular axis of the organ of Corti running from bottom to top in Y. The tonotopic dimension runs left to right in X. Modiolar side is in front, pillar side is in back. The model then rotates 360 degrees around the central axis of the inner hair cell (also called the habenular-cuticular axis or the hair cell basal-apical axis).

Movie S10. p34 central hair cell model with synapses. This 20s movie begins with the segmented central IHC in white with segmented synapses in red (ribbons), green (PDs), and blue (PSDs). The data scrolls through the model from the perspective from which the images were acquired, with the habenular-cuticular axis of the organ of Corti running from bottom to top in Y. The tonotopic dimension runs left to right in X. Modiolar side is in front, pillar side is in back. The model then rotates 360 degrees around the central axis of the inner hair cell (also called the habenular-cuticular axis or the hair cell basal-apical axis).
